# CSsingle: a unified tool for robust decomposition of bulk and spatial transcriptomic data across diverse single-cell references

**DOI:** 10.1093/nar/gkag410

**Published:** 2026-05-04

**Authors:** Wenjun Shen, Yunfei Hu, Yuanfang Lei, Hau-San Wong, Cheng Liu, Si Wu, Xin Maizie Zhou

**Affiliations:** Department of Bioinformatics, Shantou University Medical College, 515041 Shantou, China; Chaoshan Branch of State Key Laboratory for Esophageal Cancer Prevention and Treatment, Shantou University Medical College, 515041 Shantou, China; Department of Computer Science, Vanderbilt University, 37240 Nashville, United States; Department of Bioinformatics, Shantou University Medical College, 515041 Shantou, China; Department of Computer Science, City University of Hong Kong, 999077 Kowloon, Hong Kong; College of Computer Science and Technology, Huaqiao University, 361021 Xiamen, China; School of Computer Science and Engineering, South China University of Technology, 510006 Guangzhou, China; Department of Computer Science, Vanderbilt University, 37240 Nashville, United States; Department of Biomedical Engineering, Vanderbilt University, 37240 Nashville, United States

## Abstract

Accurate deconvolution of bulk and spatial transcriptomes is essential for studying tissue architecture and disease, yet remains challenged by unmodeled differences in cellular RNA content and cross-source heterogeneity. We introduce CSsingle, a unified deconvolution framework that explicitly corrects for cell-type-specific RNA content differences using either External RNA Controls Consortium (ERCC) spike-ins or a computational estimator, while robustly harmonizing data across platforms. CSsingle employs an iteratively reweighted least-squares model initialized by marker-gene sectional linearity, enabling accurate inference of cell-type proportions from diverse single-cell references. In bulk data, CSsingle outperforms existing methods by correcting systematic errors, including neutrophil underestimation in blood and tumor purity underestimation in breast tumor. Applied to spatial transcriptomics, CSsingle enables fine-grained mapping of cellular organization in the developing human pancreas and reveals functionally distinct niches in colon cancer. By integrating cell size awareness with cross-platform robustness, CSsingle advances the integrative analysis of complex tissues.

## Graphical Abstract

**Graphical Abstract:**
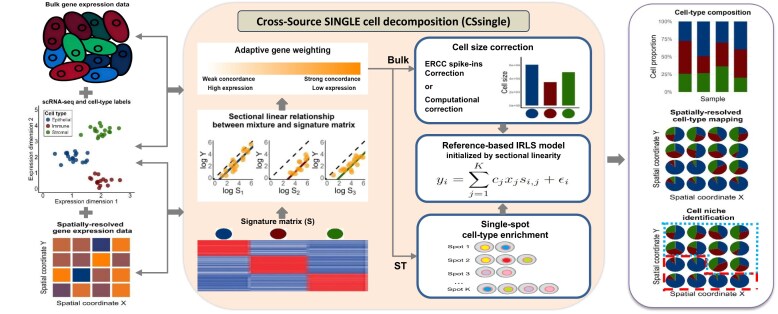

## Introduction

Deciphering cellular heterogeneity within disease-relevant tissue microenvironments is essential for identifying specific cell populations as therapeutic targets [1, 2]. While conventional bulk transcriptomics (microarrays and RNA-seq) have enabled large-scale clinical studies due to their technical simplicity and low cost, they measure only average gene expression across cell populations, masking critical cellular heterogeneity. Single-cell RNA sequencing (scRNA-seq) overcomes this limitation by offering genome-wide resolution of cellular diversity [3–5], but its high cost and requirement for high-quality tissue restrict its application in large clinical cohorts [6, 7]. Spatial transcriptomics (ST) technologies, such as 10x Genomics Visium [8] and Slide-seq [9], preserve tissue architecture while capturing whole transcriptome information, yet lack true single-cell resolution [10]. Computational deconvolution has emerged as a powerful strategy to integrate these complementary data types, enabling the inference of cellular composition from bulk and ST data using scRNA-seq references [11–13].

The utilization of a signature matrix not only improves computational efficiency but also significantly affects the accuracy of deconvolution. The construction of a signature matrix is fundamentally influenced by data normalization. While some methods [14–18] use raw read or unique molecular identifier (UMI) counts generated by scRNA-seq [19], this approach fails to account for variable sequencing depth (total number of reads or UMI molecules per cell) across cells. Alternative approaches employ depth-normalized values such as transcripts per million (TPM) or counts per million (CPM)-normalized values to mitigate this issue [20–24]. However, TPM/CPM normalization assumes equal RNA content per cell [25]. Conventional bulk transcriptomic technologies measure average gene expression across cell populations, a value confounded by cell size that is commonly referred to as the absolute cellular RNA contents. Larger cells contain higher absolute RNA content and therefore contribute disproportionately to bulk measurements. Consequently, deconvolving mixtures of cell types with substantially different sizes using TPM/CPM-normalized references yields systematically biased proportion estimates unless explicit cell size correction is applied. Despite its fundamental importance, a systematic evaluation of cell size bias in deconvolution is lacking.

Equally challenging is the correction of technical and biological disparities between mixture data and single-cell references. In bulk deconvolution, Bisque [24] learns gene-specific transformations of bulk data to correct for technical biases between the bulk and reference data. CIBERSORTx [23], an extension of CIBERSORT [20], employs a batch correction method to reduce cross-platform variation between bulk mixtures and the signature matrix. In particular, CIBERSORTx requires multiple bulk samples (optimally $\ge 10$) for stable performance. In spatial deconvolution, existing methods [26–32] have advanced the field but do not fully account for cross-source variation between ST and reference data, significantly affecting the accuracy of estimates [13]. Resolving technical and biological variations between a single bulk mixture or ST spot and the reference data remains a critical challenge, underscoring the need for deconvolution frameworks capable of robust data harmonization across diverse experimental contexts.

To address these challenges, we introduce CSsingle (Cross-Source SINGLE cell decomposition), a unified deconvolution framework for accurate and robust estimation of cell type composition from bulk and ST data. CSsingle is specifically designed to explicitly model and correct for both biological and technical variations inherent in individual mixtures and the signature matrix. Its key innovations include: (i) adaptive weighting that leverages marker-gene sectional linearity to achieve robust cross-source harmonization; (ii) integrated cell size correction that accounts for cell-type-specific RNA content differences using either External RNA Controls Consortium (ERCC) spike-ins or a novel computational estimator; and (iii) spatially resolved deconvolution enabling fine-grained dissection of tissue microenvironments at single-spot resolution. Through comprehensive evaluations across cell lines, blood, solid tissues, and both synthetic and real–world datasets (summarized in Table 1), we demonstrate that CSsingle systematically outperforms current state-of-the-art methods in scenarios involving cross–source heterogeneity. In particular, we rigorously assess the impact of cell size correction on deconvolution accuracy, showing that CSsingle effectively corrects size-induced biases in bulk data. The results underscore CSsingle’s versatility and robustness, positioning it as a powerful tool for integrative analyses of single-cell, bulk, and ST data across diverse biological and clinical contexts.

**Table 1.** Details of the data sets used in the study

Tissue type	Data source	Protocol	Data type	# Cell	# Samples	ERCC
				types		spike-ins
						availability
Cell line	GSE129240 [33]	NA	RNA-seq	2	13	Yes
Blood	GSE132044 [34]	Smart-seq2	scRNA-seq	5	14	No
		10x Chromium v2				
		10x Chromium v3				
		CEL-seq2				
		Drop-seq				
		inDrops				
		Seq-Well				
	GSE127813 [23]	NA	RNA-seq	6	12	No
	GSE107011 [35]	Smart-seq2	RNA-seq	29	127	Yes
	GSE60424 [36]	Illumina TruSeq	RNA-seq	6	114	No
	GSE73072(H3N2,SI) [37]	Affymetrix	Microarray	NA	371	No
Pancreatic islet	E-MTAB-5061 [38]	Smart-seq2	scRNA-seq	6	10 (6H+4T2D)	No
	GSE81608 [39]	Fluidigm C1	scRNA-seq	4	18 (12H+6T2D)	No
	GSE86473 [40]	Fluidigm C1	scRNA-seq	5	8 (5H+3T2D)	No
	GSE84133 [41]	inDrop	scRNA-seq	14	4 (3H+1T2D)	No
Breast cancer	GSE176078 [42]	10x Genomics	scRNA-seq	17	26	No
	TCGA-BRCA [43]	NA	RNA-seq	NA	1093	No
Mouse cortex	GSE71585 [44]	Smart-seq2	scRNA-seq	9	1414	Yes
	Allen Brain Cell Atlas [45]	Smart-seq2	scRNA-seq	9	4785	No
	10x Genomics Visium [45]	10x Visium	ST	NA	1	No
Pancreas	GSE85241 [46]	CEL-seq2	scRNA-seq	7	4	Yes
	OMIX236 [47]	10x Genomics	scRNA-seq	7	1	No
	GSE197317 [48]	10x Visium	ST	NA	7	No
Colorectal cancer	GSE178341 [49]	10x Genomics	scRNA-seq	16	181	No
	OEP00001756 [50]	10x Visium	ST	NA	1	No

## Materials and methods

### Construction of the signature matrices

Most current cell-type deconvolution techniques, which depend on a signature matrix composed of cell-type-specific GEPs, operate under the assumption that cells can be categorized into a predetermined set of types and the prevalent cell types within the bulk tissue are adequately reflected in the scRNA-seq data. To build a signature matrix from scRNA-seq data, we started with a read or UMI count matrix. We first summarized the gene counts of all cells assigned to the same cell type. The summation was followed by normalization based on total count and multiplication by a scale factor of $10^4$. This process produced a matrix of genes $\times$ cell types (denoted as $\tilde{S}$), from which a submatrix (denoted as $S$) was derived by selecting a set of differentially expressed genes. The identification of differentially expressed genes was accomplished using the FindAllMarkers function with the default parameters of the Seurat R package (version 5.0.1). Specifically, we initially isolated differentially expressed genes using a log-scale threshold of $\ge 0.25$-fold overexpression in a given cell population relative to all others. Subsequently, nonsignificant genes with a *P*-value larger than 0.01 (Wilcoxon Rank Sum test or likelihood-ratio test) were filtered out. Next, we ranked differentially expressed genes in ascending order by their *P*-values and selected the top $N$ marker genes for each cell type as the most differentially expressed marker genes. Finally, we generated multiple signature matrices by varying $N$ from 50 to 200 with step 50 by default.

### Selecting the optimal signature matrix

To determine the optimal signature matrix, CSsingle is integrated with each signature matrix to estimate the cell type proportions. The optimal signature matrix $S^{*}$ is selected as the candidate matrix whose inferred bulk/ST gene expression matrix has the highest Spearman correlation coefficient with the real bulk/ST gene expression matrix.

### CSsingle model

CSsingle is an iteratively reweighted linear regression model that decomposes the bulk or ST gene expression data into a set of predefined reference cell types to estimate cell abundances. In order to accurately estimate the cell type fraction, we designed a novel weighting scheme to properly adjust the contribution of each marker gene. We denote $\tilde{Y}$ as a cell type mixture. Both $\tilde{S}$ and $\tilde{Y}$ consist of column-normalized expression values, so that each cell type and cell type mixture have the same total count of $10^4$ across all genes shared between the scRNA-seq and cell type mixture. Additionally, we denote the truncated version of $\tilde{S}$ and $\tilde{Y}$, containing only $N$ significant differentially expressed marker genes, as $S$ and $Y$, respectively. Let $$S = {\begin{bmatrix}s_{11} & \cdots & s_{1K}\\\vdots & \ddots & \vdots \\s_{N1} & \cdots & s_{NK} \end{bmatrix}}$$ and $Y = (y_1, y_2, \ldots , y_N)^T$. The deconvolution model for an observed cell type mixture within CSsingle is defined as follows:


(1) \begin{eqnarray*}
y_i = \sum _{j=1}^K c_jx_js_{i,j} + \epsilon _i \\\epsilon _i \sim N(0, \sigma ^2)\\\forall j: x_j \ge 0 \\\sum ^K_{j=1}x_j = 1
\end{eqnarray*}

where $S$ is an $N \times K$ signature matrix that contains GEPs for the $N$ marker genes across $K$ cell types, $Y$ is an $N \times 1$ vector representing a single bulk or spatial spot GEP for the same $N$ marker genes, $X = (x_1, x_2, \ldots , x_K)^T$ is a $K \times 1$ vector containing the cell type composition from $Y$, $c_j$ denotes the cell size coefficient of cell type $j$, and $\epsilon$ models measurement noise and other possible unmodeled factors. The constraints in (Eq. 1) require the cell type fractions to be positive and to sum up to one.

To estimate $X$ and $\hat{X}$, CSsingle minimizes the weighted squared error as follows:


(2) \begin{eqnarray*}
\hat{X} = \arg \min _{X}\sum ^N_{i=1}w_i(y_i - \sum _{j=1}^K c_jx_js_{i,j})^2 \\\text{s.t } \forall j : x_j \ge 0\\\sum ^K_{j=1}x_j = 1
\end{eqnarray*}

Here, the weights $w_i$ are interdependent with both the residuals and the estimated coefficients, forming an iterative dependency loop. To address this, we employed an iterative approach known as iteratively reweighted least squares (IRLS) to solve the weighted least squares problem.

To appropriately account for the confounding effects of cell size and cross-source variation, we designed a novel adaptive weighting scheme that formulates cell type deconvolution as a minimization problem. This scheme aims to appropriately adjust the contribution of each gene, with the goal of enhancing cross-source performance and mitigating the influence of highly expressed genes in the least squares fitting procedure. To initialize an efficient and robust set of weights to solve the minimization problem, we relied on an important property of marker genes: there exists a sectional linear relationship between individual cell type mixtures and the signature matrix. To illustrate this, we examined two fundamental assumptions underlying deconvolution methods: (i) expression profiles from each cell type are linearly additive (Eq. 1), and (ii) cell-type-specific genes are exclusively or restrictively expressed in only one cell type within a cell type mixture. In an ideal scenario, genes exclusively expressed in a single cell type exhibit a strictly linear relationship with their expressions in a cell type mixture. Then Eq. 1 can be expressed as $\log y_i = u_{j^{*}} + \log s_{i,j^{*}}$, where $u_{j^{*}} = \log (c_{j^{*}}x_{j^{*}})$ and marker gene $i$ is exclusively expressed in cell type $j^{*}$. This equation highlights the ‘sectional linear relationship’ between individual cell type mixtures and the signature matrix, which enables effective management of technical and biological variations by serving as a robust foundation for gene weighting. CSsingle takes advantage of the sectional linear property to generate an efficient set of initial estimates. The weight $w_i$ for gene $i$ is initialized via the sectional linearity property of marker genes and updated iteratively during IRLS optimization. We define $\mathscr M^j$ as a finite set of marker genes for cell type $j$, where $j = 1,2,...,K$. Let $N_j = |\mathscr M^j|$ represent the total number of marker genes for cell type $j$, and consequently, $N = \sum _j N_j$. For each cell type $j$, CSsingle employs a linear regression model to fit the cell type mixture $Y$ and each cell-type-specific GEPs within signature matrix $S$ with its marker genes in log-scale using Eq. 3. The goal is to find the best-fitting curve with a regression slope of one to a data set comprising $N_j$ observations of cell-type-specific gene expression values $(\log s_{i,j})^T_{i \in \mathscr M^j}$, together with corresponding observations of the bulk or ST gene expression values of $(\log y_i)^T_{i \in \mathscr M^j}$ by minimizing the sum of the squares of the offsets of the points from the curve.


(3) \begin{eqnarray*}
\log y_i = u_j + \log s_{i,j} (i \in {\mathscr M}^j),
\end{eqnarray*}

where $u_j = \log (c_jx_j)$ is the disturbance term for cell type $j$. Therefore, we determined the estimate $u_j$ using the respective fitted linear regression model. Once $u_j$ is estimated, we then define $\delta _j = \frac{\exp {u_j}}{\sum _j \exp {u_j}}$, representing the estimated proportion of RNA content derived from cell type $j$ in $Y$, where $\exp {u_j}$ = $c_jx_j$ represents the estimated total RNA content derived from cell type $j$ in $Y$. Next, we use $\delta _j$ to generate the estimated cell-type mixture as $Y^{*} = (t_1, t_2, \ldots , t_N)^T$, where $t_i = \sum ^K_{j=1} \delta _j s_{i,j}$. Finally, the initial weight for gene $i$ is defined as:


(4) \begin{eqnarray*}
w^{(0)}_i = \frac{1}{(\left|y_i - t_i\right|^\beta \cdot t_i^{1-\beta })^2}, \text{ for } i = 1,2,...,N,
\end{eqnarray*}

Let:


(5) \begin{eqnarray*}
X^{(0)} = \arg \min _{X}\sum ^N_{i=1}w^{(0)}_i(y_i - \sum _{j=1}^K c_jx_js_{i,j})^2,
\end{eqnarray*}

(6) \begin{eqnarray*}
X^{(1)} = \arg \min _{X}\sum ^N_{i=1}w^{(1)}_i(y_i - \sum _{j=1}^K c_jx_js_{i,j})^2,
\end{eqnarray*}

where $w^{(1)}_i = \frac{1}{(\left|y_i - t_i\right|^\beta \cdot t_i^{1-\beta })^2}$ and $t_i = \sum _{j=1}^K c_jx^{(0)}_js_{i,j}$,


\begin{eqnarray*}
\cdots
\end{eqnarray*}

(7) \begin{eqnarray*}
X^{(t)} = \arg \min _{X}\sum ^N_{i=1}w^{(t)}_i(y_i - \sum _{j=1}^K c_jx_js_{i,j})^2,
\end{eqnarray*}

where $w^{(t)}_i = \frac{1}{(\left|y_i - t_i\right|^\beta \cdot t_i^{1-\beta })^2}$ and $t_i = \sum _{j=1}^K c_jx^{(t-1)}_js_{i,j}$.

The estimated coefficients converge when $\Vert X^{(t)} - X^{(t-1)} \Vert \le 0.01$, and the optimal $X^{(t)}$ is the final cell type composition estimated from the bulk data. We introduce $\beta$ to balance the relative contributions of the difference between $y_i$ and $t_i$ and the magnitude of $t_i$, with the goal of enhancing cross-platform performance and mitigating the influence of highly expressed genes in the least squares fitting procedure. The optimal $\beta ^{*}$ was selected from $\lbrace 0, 0.5, 1\rbrace$ to maximize the Spearman correlation between the inferred and real bulk/ST GEPs.

### Cell size correction in CSsingle

Within CSsingle, we introduce cell size coefficients $c_j$ to account for differences in RNA content across cell types. CSsingle implements two complementary approaches for cell size correction to address cellular RNA content heterogeneity: direct estimation from ERCC spike–ins when available, or a computational strategy when spike–ins are absent. Both correction strategies address the fundamental challenge that cells with higher RNA content contribute disproportionately to bulk RNA-seq signals, enabling CSsingle to produce proportion estimates that better reflect true cellular abundances rather than mere transcriptional contributions.

For single-cell reference datasets containing ERCC spike-in controls, we compute cell size coefficients directly from spike-in RNA counts under two key assumptions: (i) equal amounts of spike-in RNA are added to each cell, and (ii) technical effects impact spike-in and endogenous transcripts equivalently. This approach leverages spike-ins as technology-independent standards for absolute RNA quantification. Specifically, endogenous gene counts were normalized by dividing by the upper-quartile (UQ; 75th percentile by default) of the ERCC spike-in counts to adjust for technical variation across cells. We then calculated the absolute cellular RNA content for each cell by summing the normalized endogenous gene counts. The cell size coefficient for cell type $j$ is defined as the mean absolute RNA content across all cells belonging to that type.

In the absence of ERCC spike-ins, CSsingle implements a three-stage computational strategy to estimate relative cell size coefficients by systematically analyzing residual expression patterns. This approach addresses the challenge of cellular RNA content heterogeneity when direct estimations from spike-in controls are unavailable, making it particularly applicable to single-cell references lacking spike-in data. The computational correction proceeds in three stages:

*Stage 1 (screening)*: After obtaining initial proportion estimates $\hat{X}^{(0)}$ using uniform cell size factors ($c_j = 1$ for all $j$), we compute the residual vector $R = Y - S\hat{X}^{(0)}$, where $Y$ and $S$ denote the CP10K-normalized marker-gene expression profiles (GEPs) for the mixture and signature matrix, respectively. For each cell type $j$, we calculate the Spearman correlation $\rho _j$ between the residual $R$ and the cell type signature $S_j$. Cell types with absolute residual correlations exceeding a predefined threshold (|$\rho _j| > \tau$) are flagged as potentially misestimated, indicating that their expression patterns remain inadequately explained by the current model. We set $\tau = 0.25$ to focus on substantively meaningful residual signals while reducing the risk of spurious correlations. In addition, to ensure robustness and avoid amplifying noise from rare populations, we retain only cell types with initial proportions exceeding $5\%$ ($\hat{x}_j^{(0)} > 0.05$). This screening step ensures that only cell types exhibiting both evidence of potential misestimation and sufficient abundance proceed to downstream correction, thereby reducing the risk of noise-driven over-correction.

*Stage 2 (validation)*: For each candidate cell type $j^{*}$ identified in Stage 1, we evaluate whether adjusting its cell size coefficient alone improves reconstruction accuracy, measured by the Spearman correlation between inferred and real bulk GEPs. The scaled coefficients are defined as:


(8) \begin{eqnarray*}
c_j^{(\lambda ,j^{*})} = \left\lbrace \begin{array}{@{}l@{\quad }l@{}}1 + \lambda \left[1 - \exp \left(-2\dfrac{\rho _{j^{*}}-\tau }{\rho _{\max }-\tau }\right)\right], & j = j^{*},\\1, & j \ne j^{*}, \end{array}\right.
\end{eqnarray*}

where $\lambda \in \lbrace 0.5, 1.0, 1.5, 2.0, 2.5, 3.0\rbrace$ controls the magnitude of adjustment. For each value of $\lambda$, we recompute the cell type proportions and evaluate reconstruction accuracy. A cell type is retained as overestimated only if the adjusted model achieves higher reconstruction accuracy than the baseline with uniform size factors. This empirical validation ensures that only cell types with demonstrable improvement proceed to the final stage.

*Stage 3 (joint optimization)*: Let $\mathcal {O} = \lbrace j_1, j_2, \ldots , j_m\rbrace$ denote the set of cell types identified as overestimated in Stage 2. We then adjust the cell size coefficients for all cell types in $\mathcal {O}$ simultaneously using a shared scaling parameter $\lambda$:


(9) \begin{eqnarray*}
c_j^{(\lambda )} = \left\lbrace \begin{array}{@{}l@{\quad }l@{}}1 + \lambda \left[1 - \exp \left(-2\dfrac{\rho _j-\tau }{\rho _{\max }-\tau }\right)\right], & j \in \mathcal {O},\\1, & j \notin \mathcal {O}. \end{array}\right.
\end{eqnarray*}

We perform a grid search over $\lambda \in \lbrace 0, 0.5, 1.0, 1.5, 2.0, 2.5, 3.0\rbrace$ and select the value that maximizes reconstruction accuracy, yielding the final size factors $c_j^{*} = c_j^{(\lambda ^{*})}$. Due to the sum-to-one constraint on cell type proportions, adjusting any single coefficient alters the inferred abundances of other cell types. Therefore, joint optimization ensures that the final size factors are mutually consistent and globally optimal.

This computational framework provides a principled, data-driven method for correcting cell size effects without requiring external spike-in controls. By systematically analyzing residual expression patterns and optimizing size factors to maximize reconstruction accuracy, CSsingle adapts to varying cellular RNA content across cell types, enhancing deconvolution accuracy.

### Enhancing performance in microarray data and high technical variation scenarios

Given the major differences between RNA sequencing and microarray techniques, deconvolution might prove ineffective in scenarios where excessive technical variation exists. For microarray, background hybridization and probe saturation can impede the detection of transcripts at both low levels and high levels. In contrast, RNA sequencing enables the detection of a broader range of transcripts, including those with low abundance and high abundance [51, 52]. We, therefore developed a strategy for handling bulk mixtures derived from microarrays, tailored for signature matrix generated from RNA sequencing, or vice versa. We introduced an upper bound $q$, which limits the maximum value that any weight can take on. The adjusted weights are defined as:


\begin{eqnarray*}
\hat{w}_i = \left\lbrace \begin{array}{@{}l@{\quad }l@{}}w_i,\text{ if }w_i < q;\\q, \text{ otherwise.} \end{array}\right.
\end{eqnarray*}

The upper bound $q$ is selected as follows. The possible values for $q$ are defined as the $\tau ^{th}$ quantile of the gene weights $(w_1, w_2, \cdots , w_N)^T$, where $\tau$ is selected from 0.01 to 1 with a step of 0.01. For each possible value of $q$, we obtain an estimate for $X$, denoted as $\hat{X} = (\hat{x}_1, \hat{x}_2, \cdots , \hat{x}_K)^T$, by minimizing the weighted squared error with respect to the adjusted weights $(\hat{w}_1, \hat{w}_2, \cdots , \hat{w}_N)^T$:


(10) \begin{eqnarray*}
\hat{X} = \arg \min _{X}\sum ^N_{i=1}\hat{w}_i(y_i - \sum _{j=1}^K c_jx_js_{i,j})^2.
\end{eqnarray*}

Subsequently, the inferred bulk/ST GEP $\hat{Y} = (\hat{y}_1, \hat{y}_2, \cdots , \hat{y}_N)^T$ is defined as follows:


(11) \begin{eqnarray*}
\hat{y}_i = \sum _{j=1}^K c_j \hat{x}_j s_{i,j}.
\end{eqnarray*}

CSsingle calculates the Spearman correlation between the inferred and real bulk/ST GEPs, $\hat{Y}$ and $Y$. The value of $q$ corresponding to the maximum correlation coefficient is selected.

### Enrichment analysis for identifying cell types within a single spatial spot

Inspired by spatialDWLS [27], which uses PAGE [53] to identify enriched cell types in clustered regions, we developed a refined approach that integrates both absolute and relative expression metrics to detect enriched cell types at individual spatial spots. For each cell type $j$ and each spot $s$, we compute two metrics based on its marker genes. For each marker gene, we define its fold change in a spot as:


(12) \begin{eqnarray*}
F_g^{(s)} = \frac{Y_g^{(s)}}{\bar{Y}_g},
\end{eqnarray*}

where $Y_g^{(s)}$ is the expression of gene $g$ in spot $s$ and $\bar{Y}_g$ is the mean expression of gene $g$ across all spots.

For a given cell type in a spot, we then define:

1. **Absolute score**: the average of $Y_g^{(s)}$ over its marker genes.
2. **Relative score**: the average of $F_g^{(s)}$ over its marker genes.

From these per-spot scores, we compute for each cell type the median absolute score and the median relative score across all spots. A cell type is designated as *prevalent* if it meets both of the following conditions:

1. **High abundance**: its median absolute score exceeds the global $\mathrm{ 75th}$ percentile, indicating the cell type is sufficiently abundant.
2. **Consistent expression**: its median relative score is close to one, indicating that the marker genes are consistently expressed across spots.

These two criteria together capture cell types that are both highly and widely expressed throughout the tissue. For prevalent cell types, the conventional fold change relative to the mean (Eq. 12) is inadequate for detecting local enrichment because their stable and widespread expression results in fold changes near one across all spots. To overcome this, we compute an alternative fold change using the $\mathrm{ 25th}$ percentile ($Q_{1,g}$) as the reference:


(13) \begin{eqnarray*}
F_g^{(s)} = \frac{Y_g^{(s)}}{Q_{1,g}}.
\end{eqnarray*}

This lower baseline provides a more sensitive measure of local enrichment by comparing expression in each spot to the lower tail of the expression distribution. In contrast, for nonprevalent cell types, the original fold change (Eq. 12; relative to the mean) is retained.

For each spot, we calculate a $Z$-score for cell type $j$ as:


(14) \begin{eqnarray*}
Z_j^{(s)} = \frac{(f_j^{(s)} - \mu ^{(s)})\cdot n_j^{1/2}}{\sigma ^{(s)}},
\end{eqnarray*}

where $f_j^{(s)}$ is the mean fold change of marker genes for cell type $j$ in spot $s$ (computed with the appropriate reference as described in EqS. 12 and 13), $\mu ^{(s)}$ and $\sigma ^{(s)}$ are the mean and standard deviation of fold changes across all genes in spot $s$, and $n_j$ is the number of marker genes expressed for cell type $j$.

We identify enriched cell types using three complementary criteria:

1. Cell types with $Z$-scores exceeding a threshold (default $> 0$);
2. The cell type with the highest $Z$-score in each spot;
3. Cell types with spot-normalized $Z$-scores $> 0$, where normalization transforms $Z$-scores to zero mean and unit variance per spot.

The union of all three criteria determines the final set of enriched cell types per spot.

### Construction of artificial bulk data sets

The aggregated counts for the simulated bulk data set are derived from a scRNA-seq data set, with the bulk counts computed as the sum of gene counts across all cells within the same sample. Specifically, for the scRNA-seq data set spanning fewer than five samples, we generate $t$ bootstrap replicates, each of which matches the size of the original scRNA-seq data set by randomly sampling cells with replacement. Next, counts for the artificial bulk data set are generated from each bootstrap replicate by summing up gene counts from all cells within the same sample. For the artificial bulk dataset built using SMART-Seq2 single-cell profiles of peripheral blood mononuclear cells (PBMCs), we used $t = 100$. The actual cellular proportion of cell type $j$ in the sample $s$ is calculated by


\begin{eqnarray*}
\hat{x}^s_j = \frac{n^s_j}{\sum _j n^s_j}.
\end{eqnarray*}

where $n^s_j$ is the number of cells for cell type $j$ in sample $s$.

### Gene set variation analysis

Pathway gene sets were sourced from the MSigDB database (Reactome: c2.cp.reactome.v2024.1.Hs.symbols.gmt, Hallmark: h.all.v2024.1.Hs.symbols.gmt). Single-sample gene set enrichment analysis (ssGSEA), implemented via the GSVA R package, was used to calculate enrichment scores for functional terms from the Hallmark and Reactome collections. In addition, ssGSEA was employed to evaluate pathway activity in mesenchymal–acinar interactions (MAIs) within pancreas ST data.

### Functional enrichment analysis of colon niche-specific marker genes

The identification of marker genes was accomplished using the FindAllMarkers function of the Seurat R package. Specifically, we initially isolated differentially expressed genes using a log-scale threshold of $\ge 0.25$-fold over-expression in a given cell population relative to all others. Subsequently, we ranked the differentially expressed genes in ascending order by their *P*-values and selected the top 100 marker genes for each niche. The R package clusterProfiler was used to identify and visualize enriched Hallmark gene sets.

### Statistical analysis

All statistical analyses were conducted using R (version 4.4.0; available at https://cran.r-project.org/). Specific statistical tests are detailed in the figures and their respective captions when applicable. A $P$-value threshold of 0.05 was used to determine statistical significance for all tests, unless otherwise specified. Statistical significance between two groups of samples was assessed using a two-sided Wilcoxon test and reported as follows: $^{ns}P \ge .05$, $^{*}P< .05, \ ^{**}P< .01, \ ^{***}P< .001$, and $^{****}P< .0001$. The cumulative survival time was estimated via the Kaplan–Meier method, with the log-rank test from the R survminer package employed to assess survival curve disparities.

### Systematic evaluation of CSsingle and comparison against baseline methods

In this study, we benchmarked the performance of CSsingle against 11 existing methods for bulk data deconvolution: DWLS, BayesPrism, CIBERSORT, CIBERSORTx, MuSiC, MuSiC2, NNLS, SCDC, BisqueRNA, CAMmarker, and EPIC. To ensure fairness and reproducibility, all deconvolution tools were executed using their standard recommended parameters as specified in official documentation, with identical input gene expression data. Further details regarding their implementation and specific parameters can be found in the respective original publications and GitHub repositories. All parameters were initialized to their default values, unless stated differently.

- **DWLS**. We downloaded DWLS from https://bitbucket.org/yuanlab/dwls/src/default/. The signature matrix was constructed using the hurdle model in the MAST R package. In the event of negative values, the estimated proportions were adjusted to zero. When the function solve.QP fails to find a solution due to inconsistent constraints, DWLS outputs errors.
- **BayesPrism**. The R package BayesPrism was downloaded from https://github.com/Danko-Lab/BayesPrism.git.
- **CIBERSORT**. CIBERSORT was run online (https://cibersortx.stanford.edu/runcibersortx.php). No batch correction was applied.
- **CIBERSORTx**. CIBERSORTx was executed online (https://cibersortx.stanford.edu/runcibersortx.php). Batch correction was applied to reduce cross-platform variance.
- **MuSiC**. The R package MuSiC was downloaded from https://github.com/xuranw/MuSiC. MuSiC was employed with parameter ‘cell_size’ set as NULL (default value, estimating cell size coefficients from data), while MuSiC* was employed with ‘cell_size’ estimated using ERCC spike-in controls.
- **MuSiC2**. The MuSiC2 functions are available within the R package MuSiC. MuSiC2 was designed for the deconvolution of multi-condition bulk data, we thus ran it only for multi-condition bulk data. MuSiC2 was employed with parameter ‘cell_size’ set as NULL (default value, estimating cell size coefficients from data), while MuSiC2* was employed with ‘cell_size’ estimated using ERCC spike-in controls.
- **NNLS**. The NNLS method is implemented in the R package MuSiC.
- **SCDC**. The R package SCDC was downloaded from http://meichendong.github.io/SCDC. During the quality control procedure, we set the parameter ‘qcthreshold = 0.7’.
- **BisqueRNA**. The R package BisqueRNA was downloaded from https://github.com/cozygene/bisque.
- **CAMmarker**. The R package debCAM was downloaded from https://bioconductor.org/packages/release/bioc/html/debCAM.html. The parameter ‘MGlist’ defines a list of vectors, each containing known markers for one cell type. These markers are chosen from the candidate signature matrix with the lowest condition number.
- **EPIC**. The R package EPIC was downloaded from https://github.com/Gfeller-Lab/EPIC. The parameter ‘sigGenes’ defines a character vector consisting of gene names chosen to serve as a signature for the deconvolution process. These genes are selected from the candidate signature matrix with the lowest condition number.

We further benchmarked the performance of CSsingle against seven existing methods for ST data deconvolution: Seurat, RCTD, SpatialDWLS, Redeconve, SpatialDecon, SPOTlight, and SpatialDDLS. Further details regarding their implementation and specific parameters can be found in the respective original publications and GitHub repositories. All parameters were initialized to their default values, unless stated differently.

- **Seurat**. Deconvolution was performed using the FindTransferAnchors and TransferData functions from the Seurat (https://satijalab.org/seurat/) package.
- **RCTD**. The R package spacexr was downloaded from https://github.com/dmcable/spacexr. We set the parameter ‘doublet_mode’ to ‘full’ in the run.RCTD function.
- **SpatialDWLS**. Deconvolution was performed using the runDWLSDeconv functions from the Giotto (https://rubd.github.io/Giotto_site/) package. We used the signature matrix with the lowest condition number.
- **Redeconve**. The R package Redeconve was downloaded from https://github.com/ZxZhou4150/Redeconve.
- **SpatialDecon**. The R package SpatialDecon was downloaded from https://github.com/Nanostring-Biostats/SpatialDecon/.
- **SPOTlight**. The R package SPOTlight was downloaded from https://github.com/MarcElosua/SPOTlight.
- **SpatialDDLS**. The SpatialDDLS method is implemented using the R package SpatialDDLS (https://github.com/diegommcc/SpatialDDLS).

### Assessment of deconvolution performance

We assessed the performance of various deconvolution methods using Pearson’s correlation coefficient (R), root mean squared deviation (RMSD), and mean absolute deviation (mAD) as evaluation metrics. These metrics are calculated using the following equations:


(15) \begin{eqnarray*}
R = Cor(X,\hat{X})\\RMSD = \sqrt{Avg(X-\hat{X})^2}\\mAD = Avg(|X-\hat{X}|)
\end{eqnarray*}

where $X$ and $\hat{X}$ are actual and estimated cell type proportions, respectively.

## Results

### Existing deconvolution methods fail to consider differences in cell size

The deconvolution problem is typically cast in the form of a linear equation system: $\mathbf {y} = \mathbf {S} \times \mathbf {X}$, where $\mathbf {X}$ denotes the cell type composition of the cell type mixture $\mathbf {y}$, and $\mathbf {S}$ denotes a signature matrix. Computational methods aim to deconvolve cell type proportion $\mathbf {X}$ by using the signature matrix $\mathbf {S}$. To build a signature matrix, existing deconvolution methods normally use single-cell GEPs measured by read/UMI counts or TPM/CPM normalized values [20–23].

**Figure 1.**
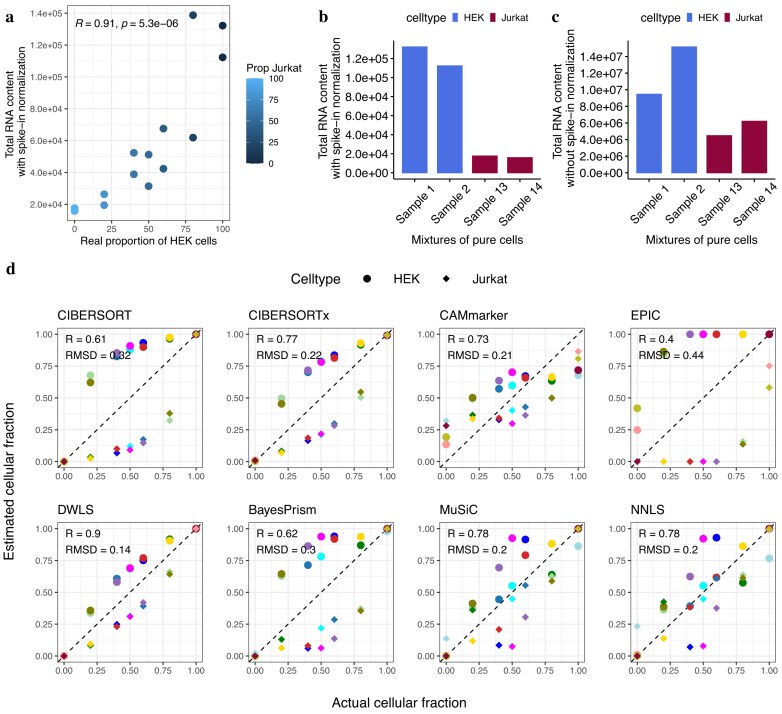
Cell type composition in mixtures of two cell types of markedly different cellular RNA contents: HEK and Jurkat cells. (**A**) Scatter plot showing Pearson correlation between absolute RNA contents and real proportions of HEK cells for bulk mixtures. (**B**) Absolute RNA contents estimated using ERCC spike-in controls for each cell type. (**C**) RNA contents estimated by summing gene counts for each cell without ERCC spike-in normalization. (**D**) Performance of representative bulk deconvolution methods on the mixtures of HEK and Jurkat cells. CIBERSORT, CIBERSORTx, CAMmarker, and EPIC (upper panels) were applied to CPM or TPM-normalized data, while DWLS, BayesPrism, MuSiC, and NNLS (bottom panels) used raw gene counts. The estimated cell type proportions are plotted against the actual cell type proportions, with shapes for cell types (circles: HEK; diamonds: Jurkat) and colors distinguishing samples.

We observed that these deconvolution methods often introduce a systematic bias in estimation when the bulk sample consists of cell types with significantly different cell sizes. To illustrate this, we considered a real data set containing ERCC spike-ins [54, 55] introduced by Zaitsev *et al*. [33], which was a compilation of 14 mixtures from two cell types (HEK and Jurkat cells) of markedly different cell sizes. ERCC spike-in controls were added to each mixture to eliminate systematic technical differences in expression between the mixtures, e.g. systematic variation introduced during the library preparations or sequencing of the samples. Each mixture in this data set, consisting of $10^6$ cells, is commonly assumed to contain equivalent quantity of messenger RNA (mRNA) molecules by existing deconvolution methods employing TPM- or CPM-normalized values. However, we observed that the absolute RNA contents of the mixtures after spike-in normalization were significantly correlated with the proportions of HEK cells (Fig. 1A), suggesting distinct cell sizes between HEK and Jurkat cells. In this data set, Samples 1, 2 and Samples 13, 14 are mixtures of pure HEK and Jurkat cells, respectively. Figure 1B indicated that HEK cells had markedly larger cell sizes than Jurkat cells after spike-in normalization. Without spike-in normalization, the estimated cell size difference is less pronounced between HEK and Jurkat cells than with spike-in normalization (Fig. 1C versus B), which is due to technical effects introduced during RNA extraction, amplification or sequencing.

For this real data set, we then used mixtures of pure HEK and pure Jurkat cells to generate cell-type-specific GEPs to construct the signature matrix. All 14 bulk mixtures were used to benchmark existing deconvolution methods against ground truth measurements. For Sample 14, DWLS failed to converge to a solution due to inconsistent constraints in the quadratic programming optimization. Consequently, only the remaining 13 samples (which yielded valid predictions across all benchmarking methods) are shown in Fig. 1D. CIBERSORT, CIBERSORTx, CAMmarker [22], and EPIC [21] constructed signature matrices by normalizing the gene counts to CPM or TPM, while DWLS, BayesPrism [17], MuSiC, and NNLS [14] used raw gene counts. As expected, all methods yielded estimated cellular fractions that systematically deviated from the actual cellular fractions (Fig. 1D). Specifically, for the deconvolution methods using a CPM- or TPM-normalized values, cellular fractions of the HEK cell type, characterized by larger cell size, were consistently overestimated. Conversely, cellular fractions for the Jurkat cell type, with smaller cell size, were consistently underestimated (Fig. 1D, top panels). The potential source of this bias in cellular fraction estimations is often overlooked because of the commonly held, though rarely explicitly stated, assumption in deconvolution that the absolute amount of total mRNA is equivalent across different cell types. Both CPM and TPM-based methods intend to correct for library size using count depth scaling and assume that all cells are initially characterized by an equivalent quantity of mRNA molecules [25]. As a result, they produce an erroneous estimation of cellular fractions due to the marked difference in cell sizes between the two cell types. Alternatively, we also tested deconvolution methods using signature matrices of raw read counts. Since these methods do not correct for bias arising from ‘technical’ library size differences, cell sizes cannot be accurately estimated. As a result, the estimated cellular fractions did not align with the actual cellular fractions (Fig. 1D, bottom panels) as well. These results highlighted the importance of incorporating cell size factors in computational deconvolution.

### An iteratively reweighted least-squares approach to robustify deconvolution estimates

**Figure 2.**
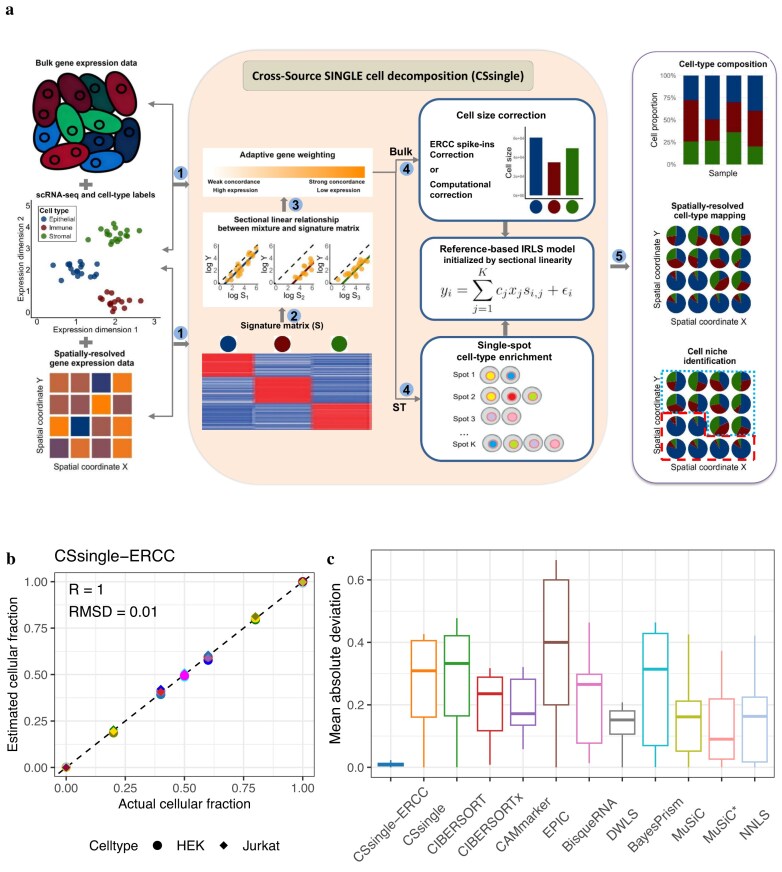
Schematic representation of the CSsingle workflow and performance validation. (**A**) CSsingle decomposes spatial and bulk transcriptomic data into a set of predefined cell types using the scRNA-seq or flow sorting reference. The main workflow is summarized in steps 1–5, each marked by a numbered circle. **(B, C)** Application of CSsingle to the deconvolution of bulk mixtures of HEK and Jurkat cells (dataset from Fig. 1). (**B**) The plot illustrates the estimated cell type proportions by CSsingle compared to the actual cell type proportions, with shapes for cell types (circles: HEK; diamonds: Jurkat) and colors distinguishing samples. (**C**) Boxplot depicting mean absolute deviation (mAD) between estimated and actual cell type proportions, with colors differentiating benchmark methods. The box encompasses quartiles of mAD, and whiskers span 1.5× the interquartile range. CSsingle–ERCC: cell–size corrected via ERCC spike–ins; CSsingle: no size correction. MuSiC*: cell_size parameter estimated using ERCC spike–ins; MuSiC: cell_size estimated from data (default).

An overview of CSsingle is shown in Fig. 2A. CSsingle addresses core challenges in cellular deconvolution through three integrated capabilities: (i) managing technical and biological variations between individual mixtures and the signature matrix by leveraging the sectional linear property of marker genes; (ii) correcting for cell size bias in bulk data by incorporating explicit size coefficients into the deconvolution model; and (iii) enabling fine–grained spatial dissection of tissue microenvironments through accurate spot–level cell type deconvolution.

To model the impact of cell size variation on deconvolution, CSsingle represents the expression of marker gene $i$ in a mixed sample $Y$ as $y_i = \sum _{j=1}^K c_jx_js_{i,j} + \epsilon _i$. Here, $x_j$ denotes the proportion of cell type $j$ in the mixture $y$, $s_{i,j}$ is the column-normalized expression level of gene $i$ in cell type $j$, $c_j$ captures the cell-type-specific RNA content (cell size), and $\epsilon _i$ represents measurement noise and other unmodeled effects. The signature matrix $S = [s_{i,j}]$ is normalized using counts per 10 000 (CP10K) to account for differences in sequencing depth, while $c_j$ explicitly corrects for cell size bias in bulk transcriptomic measurements. In CSsingle, $c_j$ is estimated from scRNA-seq data using ERCC spike-ins [54, 55], which provide technology-independent standards for absolute RNA quantification and yield cell size estimates robust to technical variability. When ERCC spike-ins are unavailable in the single-cell reference, CSsingle applies a computational estimator to infer relative cell sizes. We denote these two settings as ‘CSsingle-ERCC’ and ‘CSsingle-Comp’.

Accurate deconvolution also requires correction for technical and biological discrepancies between mixture and reference profiles. To address this, CSsingle frames deconvolution as a minimization problem solved via iteratively reweighted least squares (IRLS). While IRLS has been previously applied in deconvolution methods such as DWLS, a key innovation of CSsingle lies in its initialization using the sectional linear property of marker genes (Fig. 2A). Unlike DWLS, which initializes weights using a standard (unweighted) least–squares solution, CSsingle leverages the sectional linear relationship between individual cell type mixtures and the signature matrix to derive a biologically informed, robust initial weight set. This relationship is derived from two foundational deconvolution assumptions: (i) the linear additivity of cell type expression profiles, and (ii) the restricted expression of marker genes to specific cell types. CSsingle fits separate regression models per cell type. This cell-type-specific modeling focuses estimation on the most reliable features for each cell type, reducing interference from unrelated or noisy genes. Genes that deviate significantly from the expected sectional linearity are typically influenced by technical artifacts (e.g. platform bias or dropouts), or unmodeled biological variation (e.g. disease-state differences). By down-weighting such discordant genes during IRLS optimization, CSsingle effectively mitigates cross-source heterogeneity and enhances the robustness of cell type proportion estimates. Additionally, unlike DWLS which uses quadratic programming and cannot naturally accommodate cell size coefficients, CSsingle employs non-negative least-squares (NNLS) that directly integrates the cell size coefficients into the optimization objective. Critically, CSsingle’s NNLS solver is tightly coupled with its cell size coefficients and novel weighting scheme, enabling simultaneous correction of cell-type-specific RNA-content differences and robust harmonization of data across platforms.

To demonstrate and evaluate CSsingle-ERCC, we started with aforementioned mixtures of HEK and Jurkat cells (dataset from Fig. 1D). To leverage the sectional linear property of marker genes within real data, we first examined their linear relationships on a per-cell-type basis. Cell-type-specific GEPs for HEK and Jurkat cells were generated from pure-cell mixtures to construct the signature matrix. In Supplementary Fig. S1A, we specifically fitted a linear regression model ($\log y_i = u_j + \log s_{i,j}$) for each cell type (solid lines). In the five mixtures with varying HEK/Jurkat proportions, a strong linear relationship was observed between the HEK-specific GEPs and the bulk mixture using HEK-specific marker genes (Pearson’s $R> 0.9$; Supplementary Fig. S1A, top panels, Samples 04, 06, 08, 10 and 12). Similarly, Jurkat-specific marker genes exhibited a strong linear relationship with the bulk mixture (Pearson’s $R> 0.8$; Supplementary Fig. S1A, bottom panels). These results demonstrate the presence of a sectional linear relationship between individual bulk mixtures and the signature matrix. CSsingle-ERCC leverages the sectional linear relationship property to derive a robust and efficient set of initial weights for IRLS (Supplementary Fig. S1B). Additionally, CSsingle-ERCC employs ERCC spike-ins to estimate cell sizes for HEK and Jurkat cells (Fig. 1B). Given that HEK cells possess ∼6–7 times the RNA content of Jurkat cells, CSsingle-ERCC integrates cell size correction into its deconvolution framework, which substantially improves the accuracy of cell type proportion estimates over the uncorrected baseline (Supplementary Fig. S2A and B). Accordingly, CSsingle-ERCC provides accurate estimates of cellular fractions in various mixtures of HEK and Jurkat, significantly outperforming 10 conventional methods (Fig. 2B and C).

### CSsingle corrects for the systematic underestimation of neutrophils in blood deconvolution

Immune cells constitute a heterogeneous population with considerable diversity in size, phenotype, and function. As highlighted in systemic immune analyses, the distribution of immune cells by mass differs dramatically from their distribution by number, a consequence of pronounced size heterogeneity [56]. We then evaluated the performance of CSsingle in blood deconvolution. We constructed a signature matrix capable of distinguishing six major leukocyte subsets (B cells, Monocytes, Neutrophils, NK cells, CD4 T cells, and CD8 T cells) based on purified immune profiles generated by Linsley *et al*. [36]. This matrix was applied to a validation cohort comprising bulk RNA-seq profiles from whole blood samples of 12 healthy adults with corresponding flow cytometry measurements serving as ground-truth cell type proportions [23].

**Figure 3.**
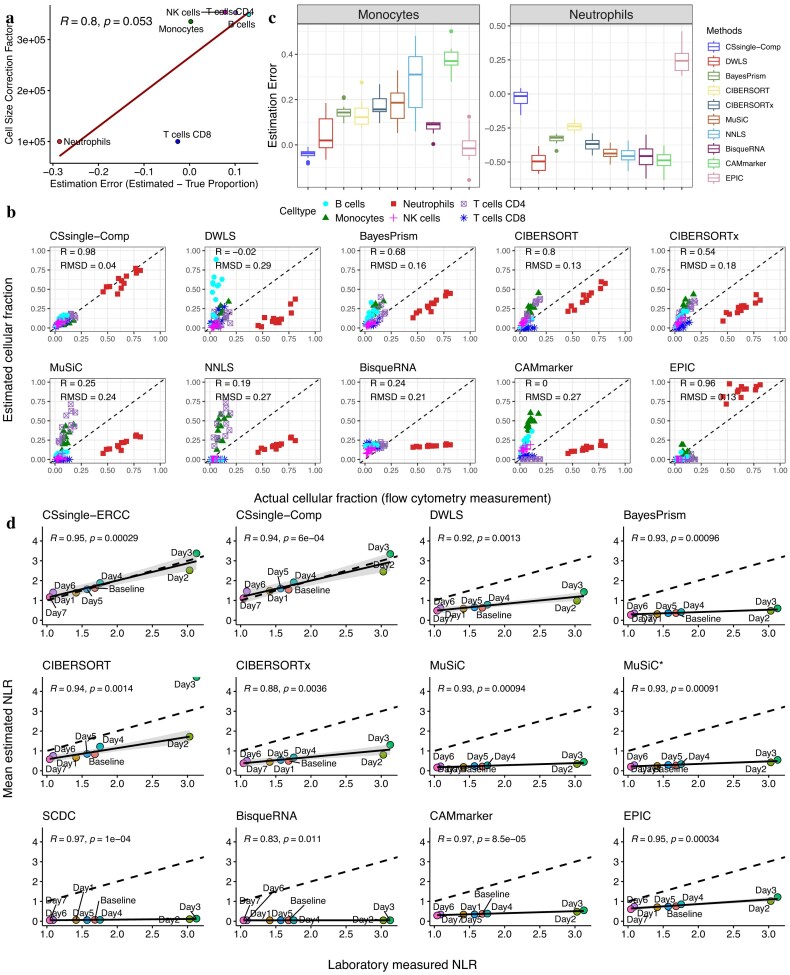
CSsingle corrects for the systematic underestimation of neutrophils in blood deconvolution. (**A**) Computational size factors correct deconvolution biases. Cell size factors estimated from computational correction are plotted against estimation errors derived without any size correction. (**B**) Decomposition benchmark on bulk RNA-seq data from whole blood samples with corresponding flow cytometry measurements serving as ground-truth cell type proportions. The plot compares estimated versus ground-truth cell type proportions, colored by cell type identity. (**C**) Comparison of estimation error for monocytes and neutrophils across deconvolution methods. Positive values indicate overestimation; negative values indicate underestimation. (**D**) Correlation between laboratory-measured and estimated neutrophil-to-lymphocyte ratio (NLR) in symptomatic infected (SI) group of influenza H3N2. Data points are labeled by days post-inoculation, with the baseline representing pre-inoculation and day 1 indicating the inoculation day. The dashed line indicates perfect concordance ($y = x$), and the solid line is a linear regression fit.

In the absence of ERCC spike-ins, CSsingle-Comp applies a computational framework that estimates cell size coefficients from endogenous gene expression. The method innovatively utilizes reconstruction residuals as an internal diagnostic for detecting cell size biases. By analyzing correlations between cell-type-specific expression profiles and reconstruction residuals, CSsingle-Comp identifies which cell types require size correction and determines appropriate scaling factors. More details are provided in the ‘Materials and methods’ section. As shown in Fig. 3A, CSsingle-Comp applied larger scaling factors to cell types that were overestimated without cell size correction, including B cells, monocytes, NK cells, and CD4 T cells. Conversely, it assigned smaller scaling factors to cell types that were otherwise underestimated, such as neutrophils and CD8 T cells. This correction pattern aligns with fundamental biological differences between these immune populations. Neutrophils and monocytes originate from a common granulocyte-monocyte progenitor, yet differ markedly in cellular volume and abundance [56]. Although neutrophils are the most abundant circulating leukocytes, their contribution to total immune cell RNA is systematically underrepresented, whereas monocytes contribute disproportionately due to their larger cell size [57, 58]. When benchmarked against ground-truth cell proportions from flow cytometry, CSsingle-Comp achieved the highest concordance (Pearson’s $R = 0.98, RMSD = 0.04$; Fig. 3B). In contrast, other methods, with the exception of EPIC, consistently underestimated neutrophil proportions and overestimated monocyte proportions (Fig. 3B). The systematic overestimation of monocytes and underestimation of neutrophils in conventional deconvolution therefore reflects the uncorrected bias introduced by differences in cellular RNA content, which scales with cell size rather than cell number (Fig. 3C).

We further benchmarked these bulk decomposition methods using real clinical samples. Specifically, we analyzed bulk transcriptomic data from a study involving influenza challenge in healthy adults aged 18–45, who were vaccinated with the A/Wisconsin/67/2005 (H3N2) strain. Genome-wide gene expression was evaluated in their peripheral blood using Affymetrix microarrays before the challenge and on days 2–7 after the challenge [37]. Prior studies have shown that NLR was associated with disease severity and mortality for influenza and Covid-19 patients [59–61]. Thus, the ability to accurately deconvolve neutrophils and lymphocytes in blood samples has significant implications in clinical applications.

To evaluate the performance of deconvolution methods, we assessed the concordance between their estimates of neutrophil and lymphocyte proportions and standard laboratory-derived measurements, with the latter obtained from a pre-existing study [62]. These laboratory-derived measurements were recorded daily from day 1 to day 7, along with a baseline measurement taken before inoculation. For deconvolution, we used the PBMC data set from an RNA-seq study [35] as the reference. This reference data set contained profiles for 110 immune cells, grouped into three major cell populations: neutrophils, lymphocytes, and non-neutrophil myeloid cells. Cell size estimates, obtained using ERCC spike-in controls, revealed significant differences among the three cell populations (Kruskal–Wallis test, $P < .0001$; Supplementary Fig. S3).

We first compared the accuracy of all 12 methods in deconvolving neutrophils and lymphocytes using blood samples from SI adults, who exhibited noticeable signs of infection. CSsingle with ERCC correction (CSsingle-ERCC) and computational correction (CSsingle-Comp) achieved the highest concordance with the laboratory measurements, correcting the systematic neutrophil underestimation and lymphocyte overestimation observed across other methods (Supplementary Figs S4 and S5). We further assessed temporal NLR dynamics, a recognized marker of influenza progression [62]. The temporal alterations in the NLR estimated by CSsingle-ERCC and CSsingle-Comp showed excellent agreement with laboratory-derived NLR measurements (Pearson’s $R = 0.95$ for CSsingle-ERCC, $R = 0.94$ for CSsingle-Comp; Fig. 3D). Although other methods also displayed positive correlations between the estimated and laboratory measured NLRs (Pearson’s $R > 0.8$, $P < .05$; Fig. 3D), their NLRs were consistently underestimated across time points. These results demonstrate that CSsingle, implemented with either ERCC or computational correction, accurately corrects both neutrophil proportions and the NLR in whole blood samples, underscoring its potential for clinical use.

### CSsingle corrects for the systematic underestimation of tumor purities in breast tumor deconvolution

**Figure 4.**
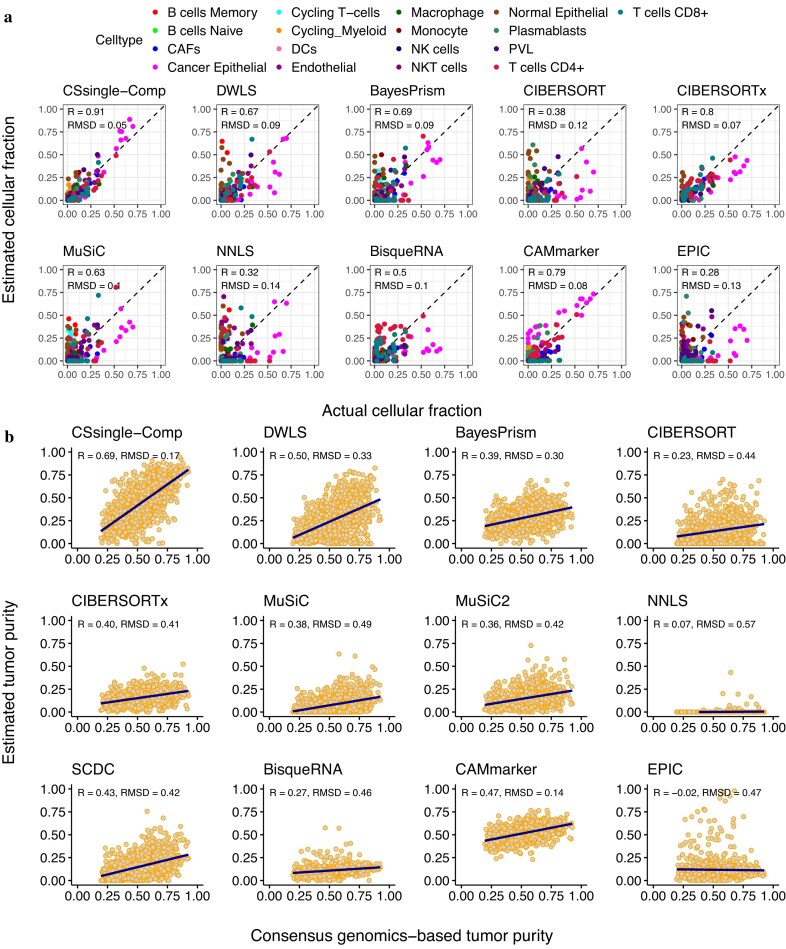
CSsingle corrects for the systematic underestimation of tumor purities in breast tumor deconvolution. (**A**) Decomposition benchmark on the simulated bulk data of breast tumor. The plot compares estimated versus ground-truth cell type proportions, colored by cell type identity. (**B**) Tumor purity estimation performance in 1093 The Cancer Genome Atlas (TCGA) breast cancer samples. Transcriptomic purity estimates from 12 deconvolution methods plotted against genomic consensus purity derived from DNA-sequencing.

The proportion of malignant cells within a tumor mass, referred to as tumor purity, is a fundamental metric in cancer research. Precise assessment of tumor purity is crucial for clinical translation, as it directly informs prognostic stratification and therapeutic response prediction [63, 64]. However, accurate deconvolution is challenged by the high heterogeneity of the tumor microenvironment (TME), which encompasses malignant, immune, and stromal components, each with distinct sizes and phenotypes. We next extended our analysis to breast tumor data, where tumors were stratified by the clinical subtypes HER2$+$, triple-negative breast cancer (TNBC), and ER$+$ [42]. To evaluate performance, we first tested CSsingle-Comp on simulated tumors reconstructed from single cells of TNBC and ER$+$ subtypes collected from Wu *et al*. [42]. Using HER2$+$ tumor cells from the same dataset as the reference, the resulting signature matrix distinguished 17 malignant and nonmalignant cell types. Evaluation on these reconstructed breast tumor samples revealed that conventional deconvolution methods, with the exception of CAMmarker, systematically underestimated the proportion of cancer epithelial cells. In contrast, CSsingle-Comp achieved the best concordance with ground-truth cell proportions (Pearson’s $R = 0.91$; Fig. 4A), followed by CIBERSORTx ($R = 0.8$) and CAMmarker ($R = 0.79$).

To extend our assessment to real clinical samples, we analyzed 1093 breast tumors from TCGA [65], using the same HER2+ tumor reference dataset employed in the simulated analyses [42]. The cancer epithelial proportion predicted by each method was taken as the tumor purity estimate. These transcriptomic predictions were compared against genomic consensus tumor purities derived from four DNA sequencing-based methods (ABSOLUTE [66], AbsCNSeq [67], ASCAT [68], and PurBayes [69]), following the approach reported in Revkov *et al*. [70]. Benchmarked against the genomic consensus purity, CSsingle-Comp achieved the highest correlation (Pearson’s $R=0.69$; Fig. 4B), followed by DWLS ($R=0.5$) and CAMmarker ($R=0.47$). In contrast, conventional deconvolution methods systematically underestimated purity in the TCGA-BRCA cohort. CSsingle-Comp mitigates this issue through integrated cell size correction, resulting in more accurate tumor purity estimates that can strengthen the clinical utility of transcriptomic data.

### CSsingle improves cross-source deconvolution

**Figure 5.**
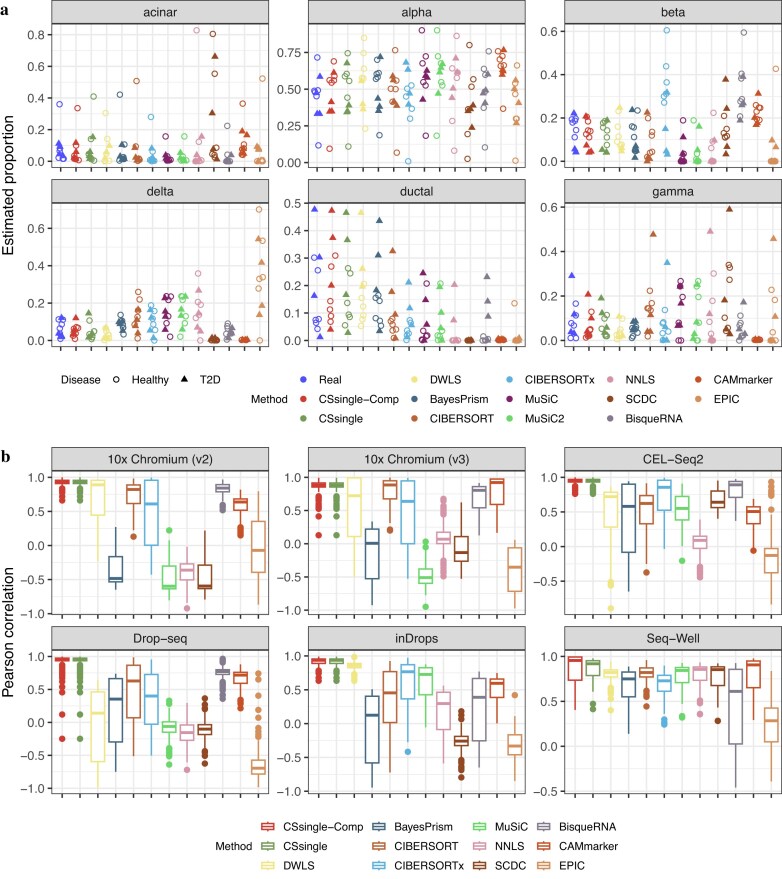
CSsingle improves cross-source deconvolution. (**A**) Jitter plots displaying true and estimated cell type proportions in pancreatic islet. Each color represents a benchmarked method. Healthy subjects are denoted as dots while T2D subjects are denoted as triangles. (**B**) Decomposition benchmark of human PBMC using scRNA-seq reference data derived from six distinct scRNA-seq methods (10x Chromium v2, 10x Chromium v3, CEL-seq2, Drop-seq, inDrops, and Seq-Well).

To evaluate CSsingle’s robustness to cross-platform variation, we further benchmarked the performance of CSsingle using an artificial pancreatic islet data set, where the bulk data and reference data were generated on distinct platforms and under varying disease conditions. Specifically, we constructed the artificial bulk pancreatic islet mixtures by aggregating single cells of six well-characterized cell types (alpha, beta, gamma, delta, acinar, and ductal) for each healthy and diseased sample from Segerstolpe *et al*., where single cells were profiled by SMART-Seq2 [38]. We constructed the signature matrix using scRNA-seq data from healthy donors, sourced from two studies: four cell types (alpha, beta, delta, and gamma) from Xin *et al*. [39] and five cell types (alpha, beta, delta, acinar, and ductal) from Lawlow *et al*. [40]. Both sets of single cells were profiled by Fluidigm C1. In this context, the signature matrix was built by integrating single cells from the two studies to obtain a more complete picture of the diverse cell types that make up the tissue. For each cell type, we observed that their specific GEPs within the signature matrix were well-correlated with the healthy or T2D bulk mixture by applying linear regression to their marker genes in log-scale via Eq. 3 ($P < .05$; Supplementary Figs S6A and S7A). This analysis revealed that the bulk mixtures remained sectional linear with the signature matrix, despite being derived from different sequencing methods and different states of the disease. However, considerable variations were observed for many genes. We therefore hypothesized that these observed variations were, to some extent, shaped by technical and biological differences between the bulk mixture and signature matrix derived from different sources. To take advantage of the good concordant genes, CSsingle generated a set of initial coefficients by up-weighting marker genes with lower expression and strong concordance and down-weighting genes with higher expression and weak concordance between the bulk mixture and signature matrix (Supplementary Figs S6B and S7B). The results showed that CSsingle achieved the best performance ($R = 0.975$, $mAD = 0.031$) over all other methods, followed by DWLS ($R = 0.952$, $mAD = 0.052$) and BayesPrism ($R = 0.938$, $mAD = 0.051$) (Supplementary Table S1). Although the signature matrix was only built from healthy samples, we found that CSsingle proved adept at accurately estimating the fractions of major and minor cell types for both healthy and T2D bulk mixtures (Fig. 5A and Supplementary Fig. S8). Notably, CSsingle-Comp performed comparably to CSsingle, suggesting that cell size bias does not significantly influence these experiments and allowing us to assess cross–source robustness without confounding from size effects.

The proliferation of diverse scRNA–seq protocols necessitates deconvolution methods that are robust to platform–specific technical variation. To benchmark different deconvolution methods in this challenging but realistic scenario, we considered a benchmark data set introduced by Ding *et al*. [34], who were among the first to systematically examine how well scRNA-seq methods captured biological information. This data set profiled human PBMCs samples employing two low-throughput plate-based methods, Smart-seq2 and CEL-Seq2, alongside five high-throughput methods: 10x Chromium v2, 10x Chromium v3, Drop-seq, Seq-Well, and inDrops. SMART-Seq2 prepares full-length libraries, which is essentially identical to the process used in bulk RNA-seq. We created artificial bulk RNA-seq data using PBMCs single cells profiled by the SMART-Seq2 protocol. Subsequently, we built signature matrices using scRNA-seq data derived from six distinct scRNA-seq methods (10x Chromium v2, 10x Chromium v3, CEL-seq2, Drop-seq, inDrops, and Seq-Well), all of which incorporate UMIs. CSsingle–Comp yielded results identical to CSsingle on five platforms and marginally improved performance on Seq–Well, suggesting that cell size bias is not a major confounder in these experiments (Fig. 5B). Across all six platforms, CSsingle consistently achieved high precision (Fig. 5B, $R = 0.821 - 0.948$; Supplementary Fig. S9, $mAD = 0.034-0.062$; Supplementary Table S2), with strong performance on challenging datasets such as Drop–seq ($R = 0.948, mAD = 0.034$) and CEL–Seq2 ($R = 0.934, mAD = 0.035$). While DWLS performed comparably with CSsingle on 10x Chromium v2 ($R = 0.904$ versus CSsingle $R = 0.859$), its accuracy dropped substantially on other platforms such as Drop–seq ($R = 0.377, mAD = 0.109$). CIBERSORT, BisqueRNA, and CAMmarker maintained moderate performance on most platforms ($R = 0.553-0.848$, $0.421-0.885$, and $0.509-0.797$, respectively), while CIBERSORTx’s performance varied substantially by platform, excelling on CEL–Seq2 ($R = 0.861$) but performing poorly on Drop–seq ($R = 0.375$). Methods including BayesPrism, MuSiC, SCDC, NNLS, and EPIC showed inconsistent or negative correlations on several platforms, indicating limited robustness to technical variation. Furthermore, CSsingle achieved the smallest RMSD and mAD for four out of five cell types (Supplementary Table S3). These results demonstrate that CSsingle maintains robust deconvolution performance across diverse experimental protocols, underscoring its utility for cross–platform integrative analyses.

Finally, to assess the effectiveness of the sectional linear initialization, we compared CSsingle against a version initialized with constant gene weights. The adaptive weighting of strongly concordant genes in CSsingle led to a significant performance improvement over the constant-weight approach, confirming the value of the sectional-linear property for robust initialization (Supplementary Fig. S10).

### CSsingle accurately estimates spatial distribution of cells from 10x visium mouse brain cortex

We extended CSsingle to ST to address the sparse and noisy nature of ST data, where low mRNA capture efficiency per spot obscures true expression signals and complicates deconvolution. We refer to this extension as CSsingle-Spatial. CSsingle-Spatial enhances ST deconvolution by detecting cell type enrichment at single-spot resolution rather than relying on pre-clustered spatial regions, amplifying cell-type-specific signals to mitigate noise (detailed in the ‘Materials and methods’ section). We first validated this approach on simulated ST data. To build a simulated ST data set, we used the CellTrek tool [45] to spatially align a well-annotated mouse scRNA-seq data set of primary visual cortex from the Allen Brain Atlas with a corresponding 10x Visium ST data set from the mouse frontal cortex [71], thereby generating a simulated ST data set with single cell resolution. We then established 200 $\times$ 200 squares to serve as simulated spots and created spot-level expression profiles by aggregating gene expression data from all cells within each square. The coordinates of the first cell in a square were designated as the spot location. For signature matrix construction, we used an independent Smart-seq2 scRNA-seq data set from the adult mouse primary visual cortex [44]. Details of each data set are shown in Table 1.

**Figure 6.**
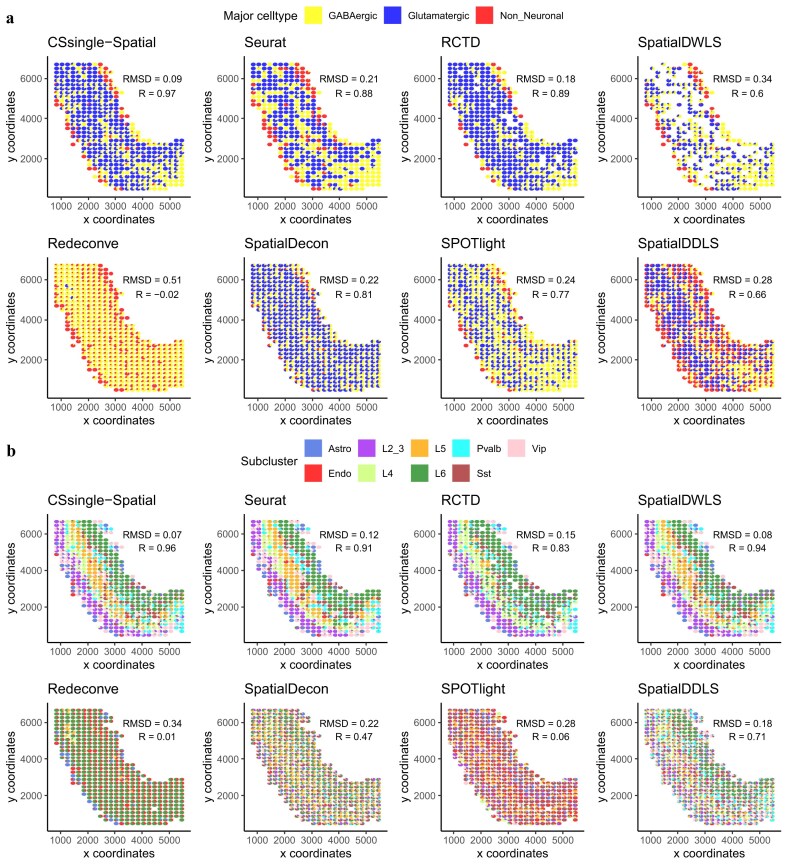
CSsingle-Spatial accurately resolves cellular composition in mouse brain. Estimated cell–type proportions are visualized as pie charts at each spatial spot for (**A**) three major cell classes and (**B**) nine neuronal subtypes.

We first applied CSsingle-Spatial to estimate the proportions of three main cortical cell types: GABAergic, glutamatergic, and non-neuronal cells, and compared its performance to leading ST deconvolution methods. The results demonstrated that CSsingle-Spatial’s predictions were highly correlated with the actual cell type distributions (Pearson’s $R = 0.97$), far exceeding the performance of seven state-of-the-art methods ($R=-0.02-0.89$; Fig. 6A; ground truth shown in Supplementary Fig. S11A). We further challenged CSsingle-Spatial to predict the spatial distribution of neuron subtypes. CSsingle-Spatial ($R = 0.96$) again achieved the best performance, followed by SpatialDWLS [27] ($R = 0.94$) and Seurat [26] ($R = 0.91$), with all three methods showing high consistency with the known locations of these subtypes across the cortex layers (Fig. 6B; ground truth shown in Supplementary Fig. S11B). In contrast, RCTD [29] displayed ectopic mapping of L4 and L6 neurons to L5, while four other methods, including Redeconve [31], SpatialDecon [30], SPOTlight [28], and SpatialDDLS [32] failed to accurately reconstruct the layered structure. Overall, CSsingle-Spatial accurately mapped both the major and minor neuronal subclusters in the cortex region, demonstrating its reliability in predicting cell type spatial distributions.

In this study, we assessed whether the incorporation of cell size coefficients, estimated by ERCC spike-ins from single-cell reference data, improves the deconvolution accuracy of CSsingle-Spatial in ST datasets. Parallel analyses were performed with and without cell size correction. Although cell sizes differed significantly among the three main clusters and nine neuronal subclusters (*P* <.001, Kruskal–Wallis test; Supplementary Fig. S12A and B), no significant difference in deconvolution accuracy was observed between the corrected and uncorrected models (Supplementary Fig. S12C and D). This finding contrasts with results in bulk RNA-seq, where cell size correction is crucial. In 2D ST data, tissue sectioning captures only a portion of each cell, leading to incomplete cellular RNA recovery and reduced sensitivity of cell size-based expression differences. Additionally, spatial complexity, such as overlapping cells and mixed cellular neighborhoods, likely overshadow the influence of absolute RNA content, diminishing the effect of cell size correction on deconvolution accuracy in spatially resolved data.

### CSsingle preserves local spatial heterogeneity in the developing human pancreas

**Figure 7.**
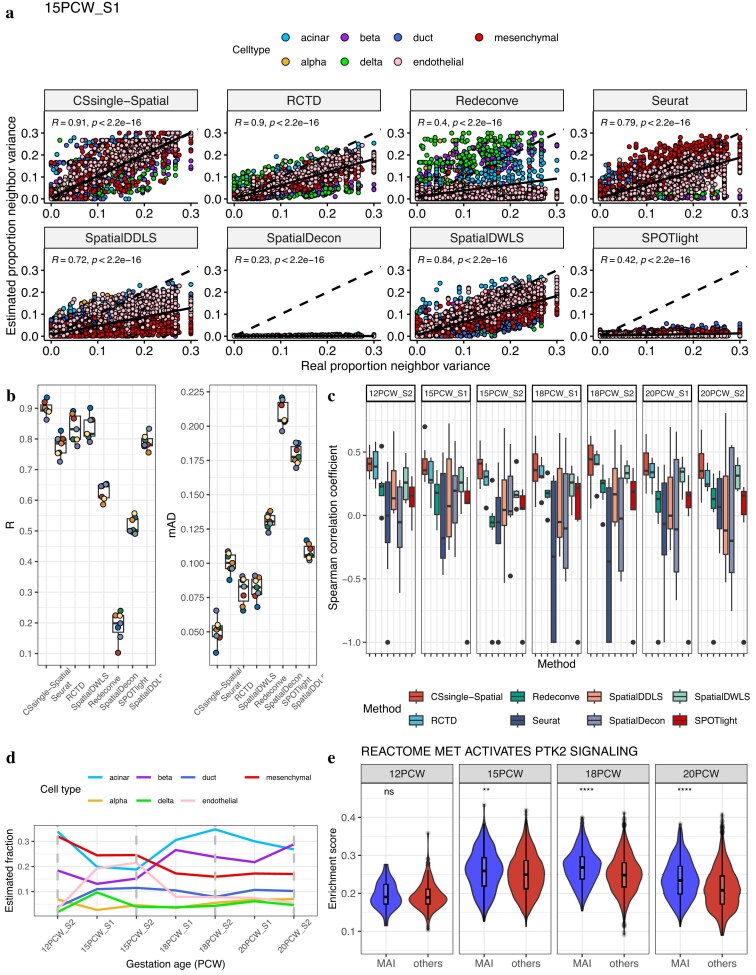
CSsingle-Spatial preserves local spatial heterogeneity. (**A**) Comparison of neighborhood variance between estimated and ground–truth cell–type proportions on simulated ST data of tissue section 1 from human fetal pancreas at 15 postconception week (PCW). Reported ‘R’ corresponds to Pearson’s correlation coefficient and *P*-values indicate the significance of these correlations. Points are colored by cell type. The dashed line indicates perfect concordance ($y = x$), and the solid line is a linear regression fit. (**B**) Decomposition benchmark in simulated ST data of human pancreas. (**C**) Spearman correlation comparison between estimated cell type proportions and cell type enrichment scores in real spatial data. (**D**) Temporal changes in mesenchymal and acinar cell abundance during pancreas development. (**E**) Reactome pathway analysis shows elevated MET-mediated PTK2 signaling in MAI-associated spots compared to non-MAI regions at 15, 18, and 20 PCWs.

To test CSsingle-Spatial’s capability to capture fine–grained spatial structure, we extended our analysis to a human fetal pancreas ST dataset. This dataset generated using 10x Visium spans four key developmental time points: 12, 15, 18, and 20 postconception weeks (PCW) [48]. We began by benchmarking CSsingle-Spatial to evaluate its accuracy in estimating the spatial distribution of cell types using simulated ST data of the human pancreas. To construct the simulated ST dataset, we aligned the 10x Genomics scRNA-seq data of human adult pancreas [47] with 10x Visium ST data from seven human fetal pancreas slides [48] using CellTrek, thus generating a simulated ST data set with single cell resolution. Subsequently, we established 150 $\times$ 150 squares to serve as simulated spots and created spot-level expression profiles by aggregating gene expression data from all cells within each square. To build the signature matrix, we used an independent scRNA-seq dataset of the human fetal pancreas at 12 PCW, generated using CEL-seq2 [46]. We evaluated local deconvolution consistency by comparing neighborhood variance between estimated and ground–truth cell–type proportions. For each ST slide, we identified the six nearest neighbors per spot and calculated the variance of cell–type proportions within each neighborhood. Across seven pancreatic ST slides, CSsingle-Spatial showed high correlation with true neighborhood variances, while other methods systematically underestimated local heterogeneity (Fig. 7A and Supplementary Figs S13–S15). The results confirm that CSsingle-Spatial preserves biologically relevant spatial variability while other methods tend to oversmooth spatial patterns, directly reflecting CSsingle-Spatial’s superior ability to capture fine–grained spatial structure. Benchmarking on seven simulated ST slides demonstrated that CSsingle-Spatial achieved the highest correlation with actual cell-type distributions (mean Pearson’s $R = 0.90$, mean $mAD = 0.05$), outperforming seven state-of-the-art ST deconvolution methods (mean Pearson’s $R = 0.19 - 0.84$; mean $mAD = 0.08 - 0.21$, Fig. 7B). Notably, despite significant differences in cell size across the seven pancreatic cell types (*P* <.001, Kruskal–Wallis test; Supplementary Fig. S16A), applying the same cell size coefficients (estimated from ERCC spike–ins) uniformly across all spatial spots again yielded no significant improvement in spatial deconvolution accuracy for the pancreatic dataset (Supplementary Fig. S16B), consistent with our earlier findings in brain ST data and confirming its limited impact on spatial deconvolution accuracy. We therefore performed all subsequent ST analyses without cell size correction.

We next applied CSsingle-Spatial to decompose cell types in the real human pancreas ST data set [48]. In the absence of ground truth labels, predictions were validated using two complementary approaches: visual agreement with classical and well-established marker-gene expression patterns (mesenchyme: COL1A1; alpha: GCG; beta: INS; delta: SST; acinar: PRSS1; duct: KRT19; endothelial: ESAM [46]) and correlation with ssGSEA (single-sample gene set enrichment analysis) enrichment scores derived from the top 50 marker genes per cell type. The spatial distribution of cell types predicted by CSsingle-Spatial aligns closely with classical marker gene expression patterns (Supplementary Figs S17–S23). Moreover, CSsingle–Spatial achieved the highest correlation with ssGSEA enrichment scores in four of seven slides and the second–highest in the remaining three, demonstrating superior and consistent performance across all slides compared to seven benchmark methods (Fig. 7C). To investigate developmental dynamics, we analyzed cell type abundances across four developmental stages. Our analysis reveals that mesenchymal cells were predominant at 12 and 15 PCW, while acinar cells became the dominant population at 18 and 20 PCW. The abundance of mesenchymal cells decreased sharply from 24% at 15 PCW to 17% at 18 PCW, whereas the abundance of acinar cells increased from 19% at 15 PCW to 33% at 18 PCW (Fig. 7D). This reciprocal shift likely reflects changes in epithelial proliferation or the development loss of mesenchymal cells. Consistent with our findings, prior studies have established that mesenchymal-epithelial interactions promote epithelial proliferation migration during organogenesis [72, 73].

To further investigate the interaction between mesenchymal and epithelial cells deconvoluted by CSsingle-Spatial, we analyzed this real ST data set to identify spatially adjacent MAIs. These interactions were significantly elevated at 15–20 PCW (Supplementary Fig. S24A). To identify pathways associated with MAI, we performed an ssGSEA analysis using Hallmark and Reactome gene sets [74] from the Molecular Signatures Database. Analysis of the Hallmark gene sets revealed a significant enrichment of the sets of target genes of epithelial-to-mesenchymal transition (EMT) and MYC in MAI, with enrichment scores notably higher in MAIs compared to other spots at 15, 18, and 20 PCW (Supplementary Fig. S24B). Furthermore, analysis of the Reactome pathways revealed that MET-mediated activation of the PTK2 (Focal Adhesion Kinase) signaling pathway was significantly enriched in MAIs compared to other spots at 15, 18, and 20 PCW (Fig. 7E). Together, these findings highlight the critical role of MAIs in activating pathways linked to mesenchymal-epithelial transition, MYC-driven transcriptional regulation, cell adhesion and proliferation, supporting their contribution to tissue remodeling during pancreatic development.

### CSsingle stratifies spatially meaningful niches in tumor colon tissue

**Figure 8.**
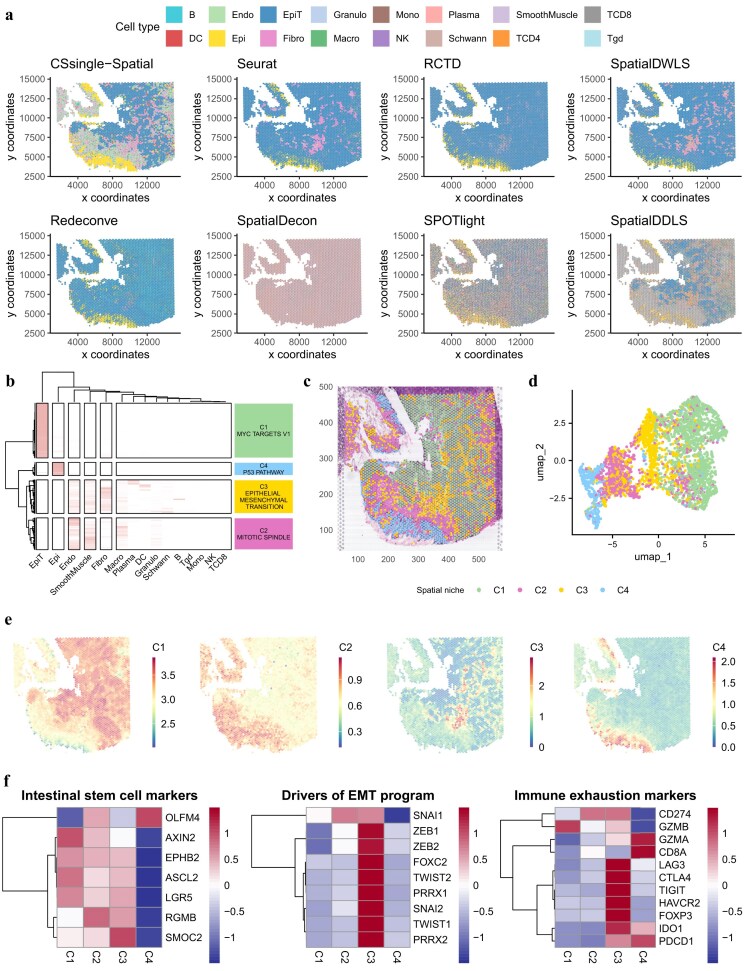
CSsingle-Spatial delineates four spatially distinct niches within the colon cancer TME. (**A**) Decomposition benchmark on tumor colon tissue. (**B**) Hierarchical clustering of cellular compositions estimated by CSsingle-Spatial segregated spatial spots into four niche categories, with enriched hallmark gene sets indicating functional specialization. (**C**) The spatial niche map. (**D**) UMAP visualization of four spatial niches identified by CSsingle-Spatial. (**E**) Feature plots of aggregated top 25 niche-specific upregulated genes reveal coordinated co-expression patterns. (**F**) The expression of well-established intestinal stem cell (ISC) markers (*left*), EMT transcription factors (*middle*) and immune exhaustion markers across niches (*right*).

We next evaluated CSsingle-Spatial’s ability to stratify spatially and functionally distinct niches in colon cancer TME. To regionally dissect spatial heterogeneity within the colon cancer TME, we deconvolved ST spots using 10x Visium data from a primary colon tumor tissue sample (ST-P1) [50]. To build the signature matrix, we used an independent scRNA-seq dataset of human colorectal cancer, generated using 10x Genomics [49]. The spatial distributions predicted by CSsingle-Spatial and other methods are shown in Fig. 8A. Hierarchical clustering of CSsingle-Spatial-estimated cellular abundances delineated four spatially distinct niches, each characterized by different cellular components (Fig. 8B): C1 (malignant-dominant), C2 (vascular/stromal-dominant), C3 (fibroblast-dominant), and C4 (normal epithelial-dominant). A detailed spatial niche map (Fig. 8C) illustrates the tissue architecture in colon cancer. Malignant-dominant (C1) and fibroblast-dominant niches (C3) exhibit spatial confinement. Normal epithelial-dominant niches (C4) are predominantly located in the periphery of the tissue, while vascular/stromal-dominant niches (C2) serve as a critical interface between C4 and tumor-driven stromal invasion (C1/C3). The UMAP (Uniform Manifold Approximation and Projection) embeddings showed clear segregation of C1, C3, and C4 into distinct groups, with partial overlap between C2 and C3, indicating transcriptomic similarity between these stromal-dominant niches that align with their spatial adjacency in tissue (Fig. 8D). Aggregation of the top 25 niche-specific upregulated genes demonstrated strong spatial co-expression patterns, confirming the biological coherence of each niche and revealing distinct spatial expression domains that further delineate functional subregions within the TME (Fig. 8E). These findings show that CSsingle-Spatial resolves niches with both spatial cohesion and transcriptomic coherence.

Functional annotation analyses of niche-specific upregulated genes revealed distinct pathway activations across the niches (Fig. 8B). Normal epithelial-dominant niches (C4) showed upregulation of genes associated with the p53 pathway. In contrast, malignant-dominant niches (C1) exhibited elevated expression of genes associated with the MYC targets v1, while fibroblast-dominant niches (C3) showed upregulation of genes involved in EMT, highlighting their complementary roles as drivers of tumor progression within the colon cancer TME. Located adjacent to tumor and epithelial regions within the lamina propria, the C2 niches displayed elevated expression of mitotic activity genes, indicative of proliferative vascular remodeling. Further analysis revealed consistent upregulation of ISC signature genes (AXIN2, EPHB2, ASCL2, LGR5, RGMB, SMOC2) across the C1, C2, and C3 niches, indicating activation of stem-like molecular programs in both malignant and stromal compartments (Fig. 8F). Critically, C3 uniquely co-upregulated EMT, immune exhaustion, and stemness signatures, indicating a fibroblast-rich niche that may drive tumor plasticity and immunosuppression. These results demonstrate that CSsingle-Spatial uniquely captures fine-scale spatial organization and functionally specialized niches within complex TMEs.

Among the benchmarked methods, Seurat and SpatialDWLS also identified spatial confinement of malignant-dominant niches (C1) and fibroblast-dominant niches (C3), while Seurat, SpatialDWLS, RCTD, and Redecovne detected normal epithelial-dominant niches (C4) (Fig. 8A). Critically, only CSsingle-Spatial resolved the vascular/stromal-dominant niche (C2), which remained undetected by all other approaches. Collectively, these results demonstrate that CSsingle-Spatial’s superior niche resolution compared to existing deconvolution approaches.

### Evaluation of signature matrix robustness

The construction of a signature matrix is a process of feature selection. To achieve this, we first identified differentially expressed genes between each cell type and all other cell types based on the thresholds of an FDR adjusted *P*-value of <0.01 and a $\mathrm{ log}_2$ mean fold change >0.25. Next, for each cell type, we ranked the differentially expressed genes by their *P*-values and selected the top $N$ genes to build the signature matrix. In this study, the value of $N$ ranged from 50 to 200, increasing by 50 at each step. We selected the optimal $N$ for constructing a robust signature matrix by maximizing the Spearman correlation between the inferred and real bulk gene expression matrices (see the ‘Materials and methods’ section). Traditionally, the stability of a signature matrix has been evaluated using the two norm condition number, and the signature matrix with the minimum condition number was retained [15, 20]. Here, we explored whether these two strategies achieved consistent results by integrating CSsingle with a signature matrix generated from each. We systematically evaluated the deconvolution results on six artificial bulk RNA-seq data sets of PBMC studied in Fig. 5B. The results showed that CSsingle exhibited comparable performance using both strategies for building the optimal signature matrix (Supplementary Fig. S25), highlighting the robustness and flexibility of CSsingle in handling different matrix construction approaches.

Furthermore, since computational efficiency and deconvolution accuracy also depend on the step size used in generating the final signature matrix, we conducted an additional experiment. We assessed the runtime and deconvolution accuracy as the step size decreased from 50 to 1. On six data sets, a step size of 50 achieved performance comparable to a step size of 1 while significantly improving computational efficiency and robustness (Supplementary Table S4).

## Discussion

Accurate decomposition of cell type mixtures is critical for studying cellular heterogeneity in clinical studies and spatial biology research. In this study, we introduce CSsingle, a unified deconvolution framework that overcomes two persistent barriers in the field: (i) the systematic bias introduced by variations in cellular RNA content, and (ii) the harmonization of data across diverse platforms and biological sources.

A core contribution of CSsingle is its explicit correction for cell size bias, a source of systematic error critically overlooked in conventional deconvolution. A common assumption prevalent in transcriptomic analysis, including cell type decomposition, is that most genes exhibit stable expression across cells, implying that RNA content remains constant across cell types. However, substantial evidence from many studies indicates frequent violations of this assumption [75, 76]. Our results demonstrate that violating this assumption leads to predictable, systematic errors. CSsingle overcomes this by incorporating cell size coefficients derived from ERCC spike-ins or, in their absence, a novel computational correction approach based on reconstruction residuals. The practical impact of cell size correction is demonstrated in two critical biological contexts where conventional methods fail. First, in blood deconvolution, conventional methods like CIBERSORT, DWLS, and MuSiC consistently underestimate neutrophil proportions while overestimating monocytes. Neutrophils, the most abundant leukocytes, typically have a smaller cellular RNA content compared to monocytes, leading to their systematic under-representation in size-unaware deconvolution. CSsingle’s explicit size correction successfully resolves this proportional bias, yielding neutrophil and monocyte estimates that align closely with flow cytometry and enabling an accurate prediction of the clinically relevant NLR. Second, in solid tumor analysis, we observed a parallel systematic underestimation of tumor purity in breast cancer samples. Tumor purity is a fundamental confounder in cancer genomics that affects the interpretation of therapeutic response biomarkers. Malignant epithelial cells often exhibit distinct RNA content compared to the surrounding stroma and immune infiltrate. By accounting for variations in cellular RNA content, CSsingle produced tumor purity estimates that showed superior concordance with genomic consensus measures for TCGA-BRCA, a crucial advancement for prognostic and therapeutic studies where accurate tumor purity is paramount.

In contrast to bulk RNA-seq, applying uniform cell size correction across all spatial spots did not significantly improve deconvolution accuracy in ST. These results may be attributed to the dominant influence of incomplete cells captured by 2D slides and partial cell overlaps in ST data, which can obscure the impact of cell size differences. Additionally, the limited resolution of spatial platforms may render cell size adjustments inadequate at the spot level. Instead, CSsingle-Spatial’s advantage lies in its ability to preserve and resolve local heterogeneity through single-spot enrichment analysis, avoiding the smoothing inherent in region-based-enriched or nonenriched deconvolution approaches. This is illustrated in the developing human pancreas, where CSsingle-Spatial maintained high correlation with true neighborhood variance, capturing fine-grained spatial patterns that other methods obscured. Furthermore, in complex tissue architectures like colon cancer, this sensitivity enabled the identification of spatially and functionally distinct niches that were obscured by other approaches. These fine-scale analyses demonstrate CSsingle-Spatial’s potential to advance our understanding of cellular interactions and tissue architecture, particularly within complex and heterogeneous microenvironments.

Deconvolution is further challenged by the need to integrate data from diverse experimental platforms and biological conditions. CSsingle addresses this through its robust cross-source harmonization, which leverages sectional-linearity initialization and adaptive weighting to mitigate technical and biological biases between reference and mixture data. Benchmarks on pancreatic islet and PBMC datasets generated with multiple single-cell protocols confirm that CSsingle maintains high accuracy across platforms, enabling reliable cell-type inference irrespective of sequencing technology or disease context. This versatility underscores CSsingle’s broad applicability in integrative transcriptomic analysis.

## Supplementary Material

gkag410_Supplemental_File

## Data Availability

CSsingle is freely available through GitHub (https://github.com/wenjshen/CSsingle) and figshare (https://doi.org/10.6084/m9.figshare.29442332). All data analyzed in this study are publicly available through online sources. Accession numbers and reference links to all data sources are presented in Table 1.
