# Supplementary material for: CSsingle: a unified tool for robust decomposition of bulk and spatial transcriptomic data across diverse single-cell references
Source: Nucleic Acids Res. 2026 May 4;54(8):gkag410. doi: 10.1093/nar/gkag410 (PMC13136905; doi:10.1093/nar/gkag410)
Supplement: gkag410_Supplemental_File [file gkag410_supplemental_file.pdf]

Nucleic Acids Research, YEAR, , 1–27
DOI added during production
Published: Date added during production
Paper

Supplementary Information

Wenjun Shen,[1,2] Yunfei Hu,[3] Yuanfang Lei,[1] Hau-San Wong,[4] Cheng Liu,[5,*] Si Wu[6,*] and Xin Maizie Zhou[3,7,*]

[1]Department of Bioinformatics, Shantou University Medical College, 515041, Shantou, China
[2]Chaoshan Branch of State Key Laboratory for Esophageal Cancer Prevention and Treatment, Shantou University Medical College, 515041, Shantou, China
[3]Department of Computer Science, Vanderbilt University, 37235, Nashville, USA
[4]Department of Computer Science, City University of Hong Kong, 999077, Kowloon, Hong Kong
[5]College of Computer Science and Technology, Huaqiao University, 361021, Xiamen, China
[6]Department of Computer Science, South China University of Technology, 510006, Guangzhou, China
[7]Department of Biomedical Engineering, Vanderbilt University, 37235, Nashville, USA
[*]Corresponding author. chengliu10@gmail.com, cswusi@scut.edu.cn, maizie.zhou@vanderbilt.edu

Received: Date Month Year. **Revised received:** Date Month Year. **Accepted:** Date Month Year
© The Author(s) YEAR. Copyright and licence statements to be updated by the publisher during production.

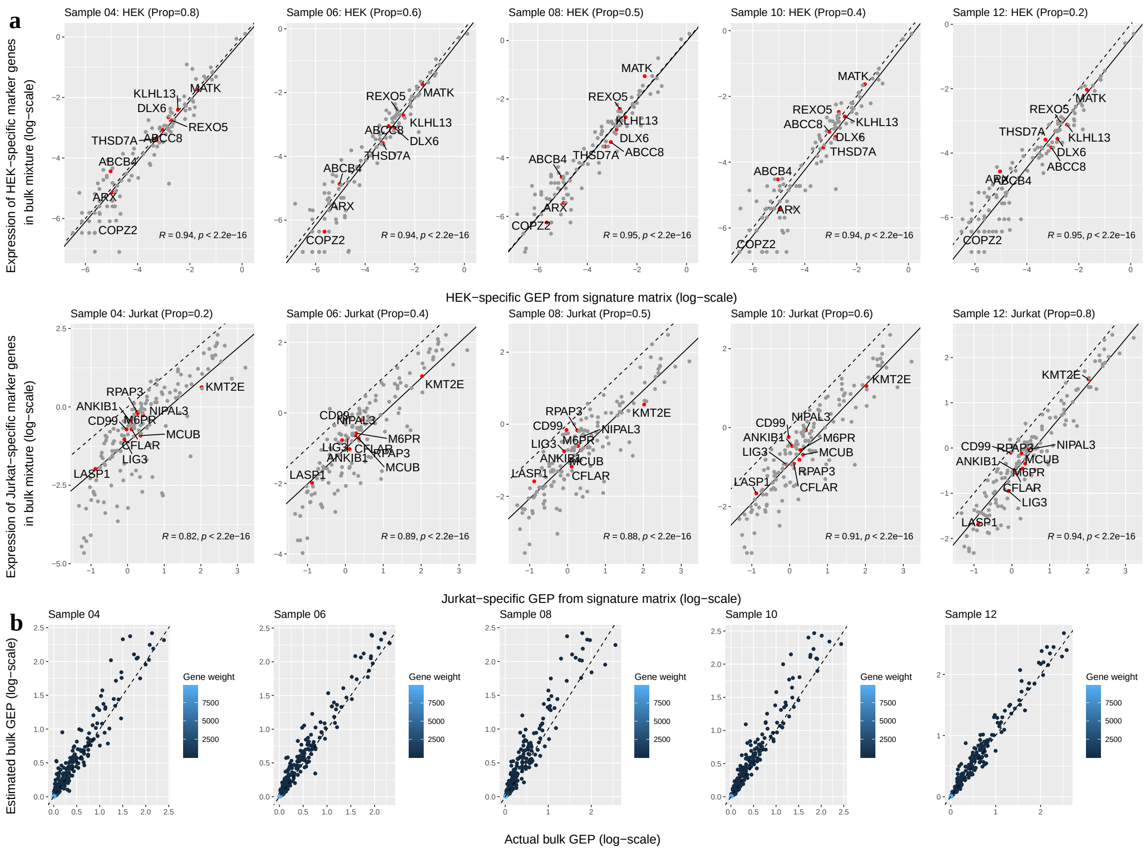

Figure S1 Sectional linear relationship between individual bulk mixtures of HEK and Jurkat cells and the signature matrix. a Linear regression with a slope of one for individual bulk mixtures and the cell type-specific GEPs for samples 04, 06, 08, 10, and 12. Top row: Linear regression between mean expression levels of HEK-specific marker genes in HEK-specific GEPs and their expression in each bulk mixture. Bottom row: Linear regression between mean expression levels of Jurkat-specific marker genes in Jurkat-specific GEPs and their expression in each bulk mixture. The dashed line in each plot represents the line of $y = x$. The signature matrix was constructed by selecting the top 150 marker genes for each cell type. Gene symbols of the top 10 most significant marker genes were plotted. **b** Scatter plots comparing estimated and actual bulk GEPs, colored by gene weights. The dashed line in each plot represents the line of $y = x$.

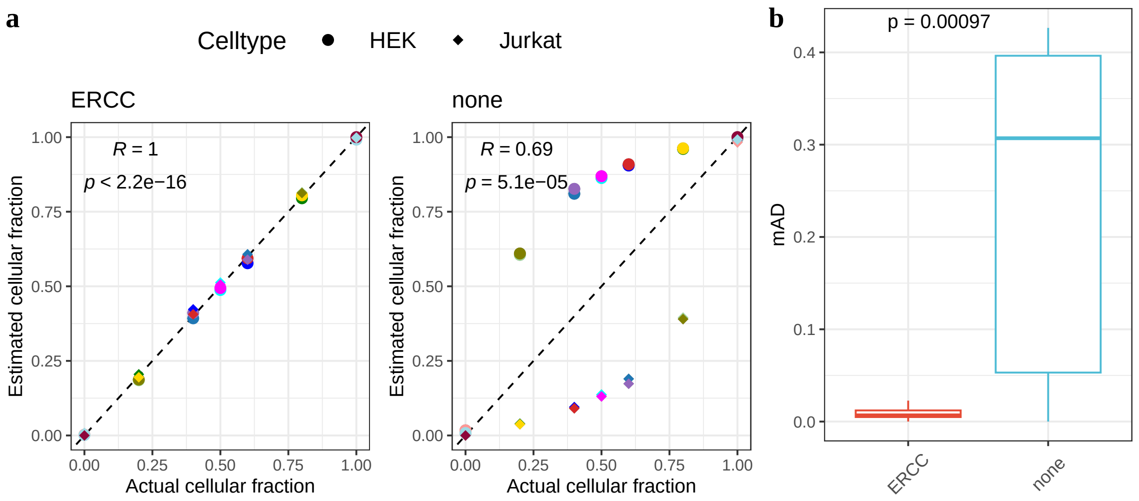

Figure S2 CSsingle deconvolution performance in HEK and Jurkat cell mixtures: with versus without cell size correction. a Plots show the Pearson correlation between estimated and actual cell type proportions with (left) and without (right) cell size correction. Shapes represent for cell types (circles: HEK; diamonds: Jurkat) and colors distinguish samples. **b** Comparison of deconvolution performance with versus without cell size correction in terms of mAD.

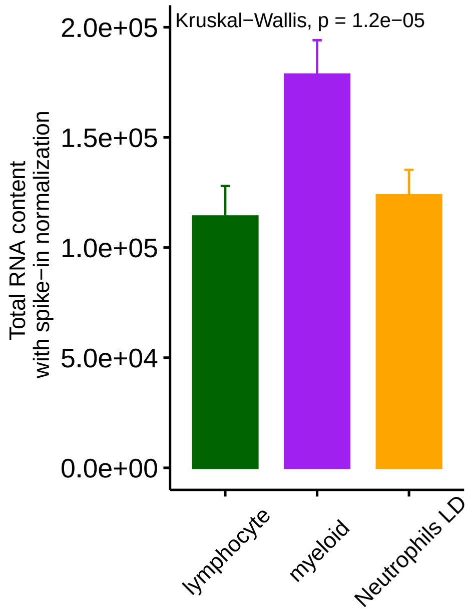

Figure S3 Comparison of the estimated cell sizes of three large immune cell groups. Statistical significance was assessed using the Kruskal-Wallis test.

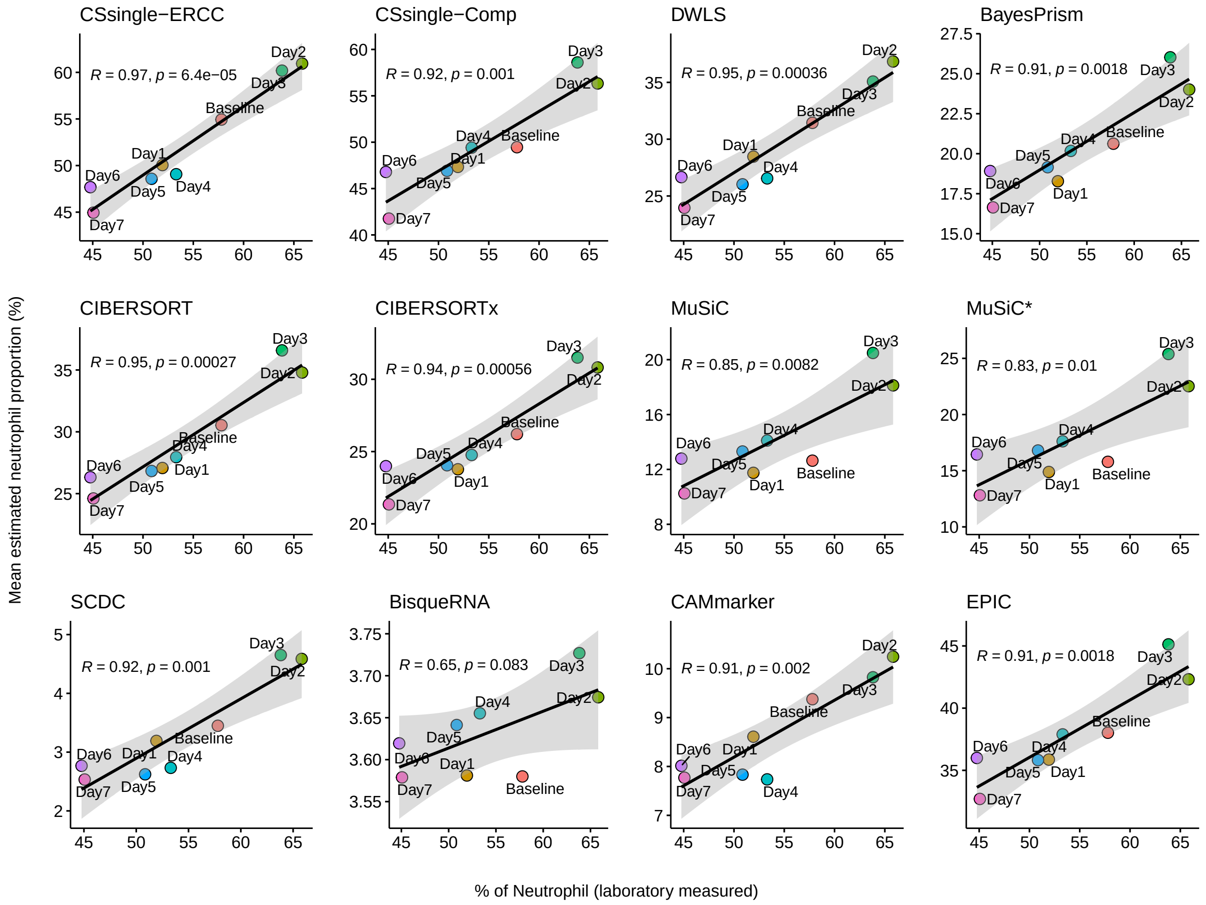

Figure S4 Correlation between laboratory measured and estimated proportions of neutrophil in SI group of influenza H3N2. Data points are labeled by days post-inoculation, with the baseline denoting pre-inoculation and day 1 marking the day of inoculation. Reported 'R' corresponds to Pearson correlation and p-values indicate the significance of these correlations.

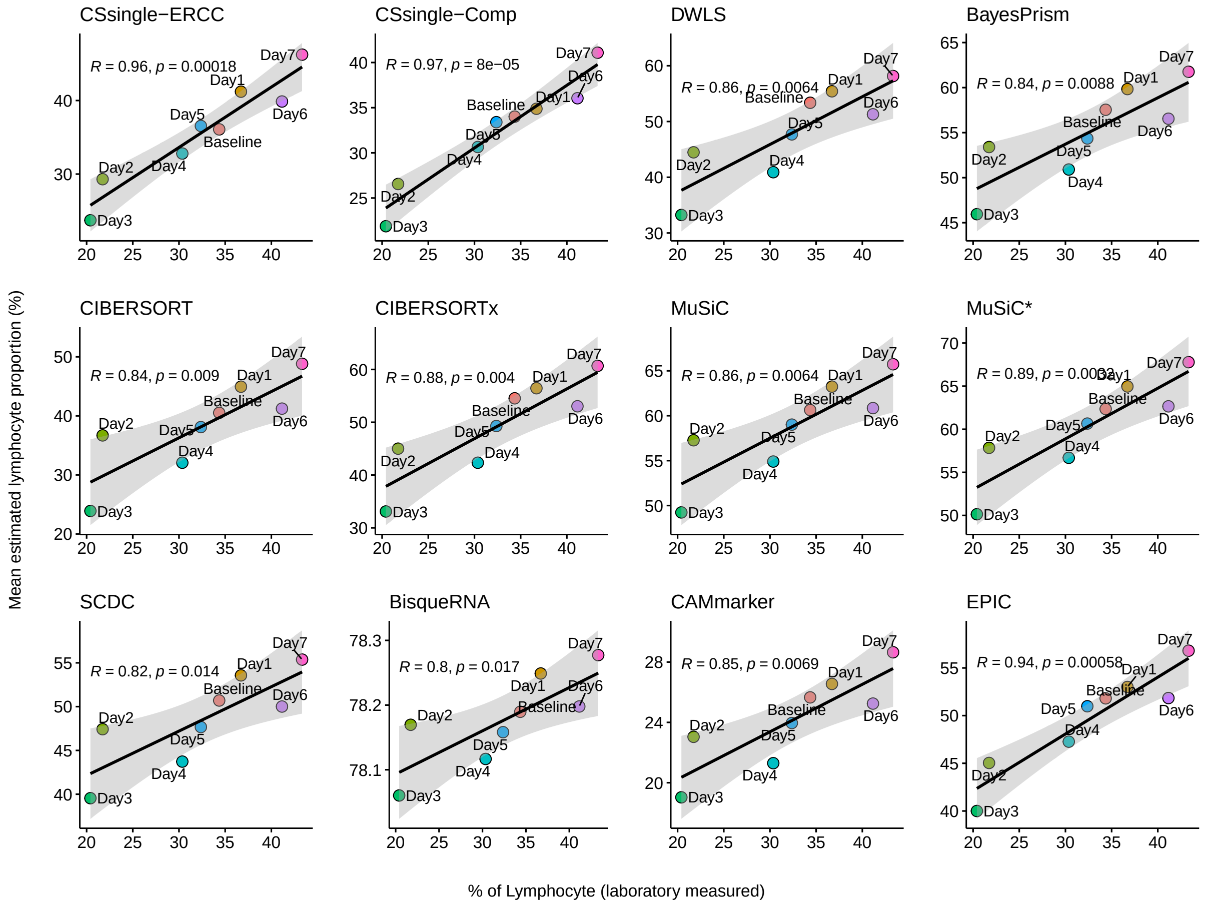

Figure S5 Correlation between laboratory measured and estimated proportions of lymphocyte in SI group of influenza H3N2. Data points are labeled by days post-inoculation, with the baseline denoting pre-inoculation and day 1 marking the day of inoculation. Reported 'R' corresponds to Pearson correlation and p-values indicate the significance of these correlations.

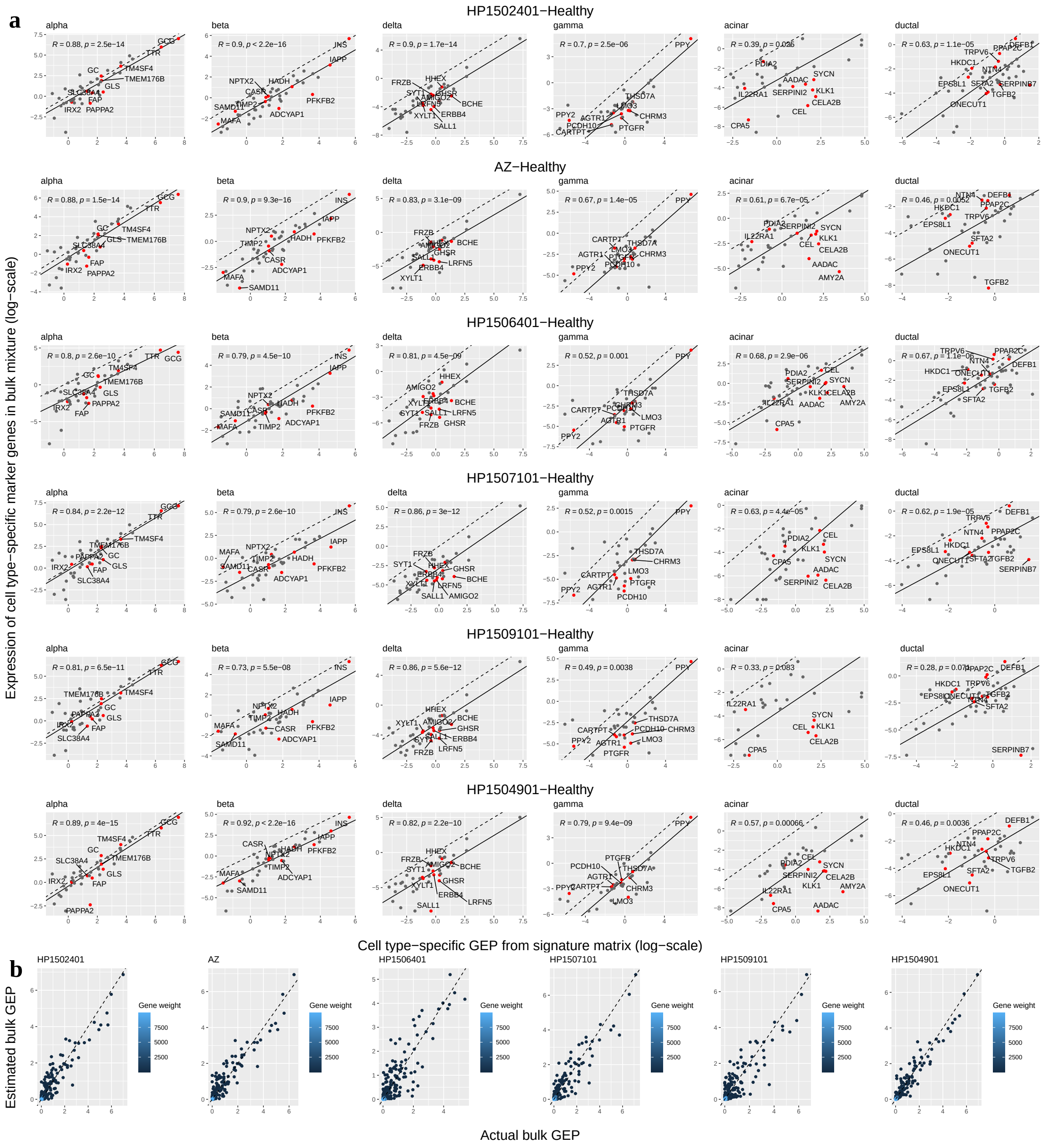

Figure S6 Sectional linear relationship between individual bulk mixtures and the signature matrix for healthy samples in the human pancreatic islet data set. a Linear regression with a slope of one for individual bulk mixtures and the cell type-specific GEPs for healthy pancreatic islet samples. The dashed line in each plot represents the line of $y = x$. The signature matrix was constructed by selecting the top 150 marker genes for each cell type. Gene symbols of the top 10 most significant marker genes were plotted. **b** Scatter plots comparing estimated and actual bulk GEPs, colored by gene weights. The dashed line in each plot represents the line of $y = x$.

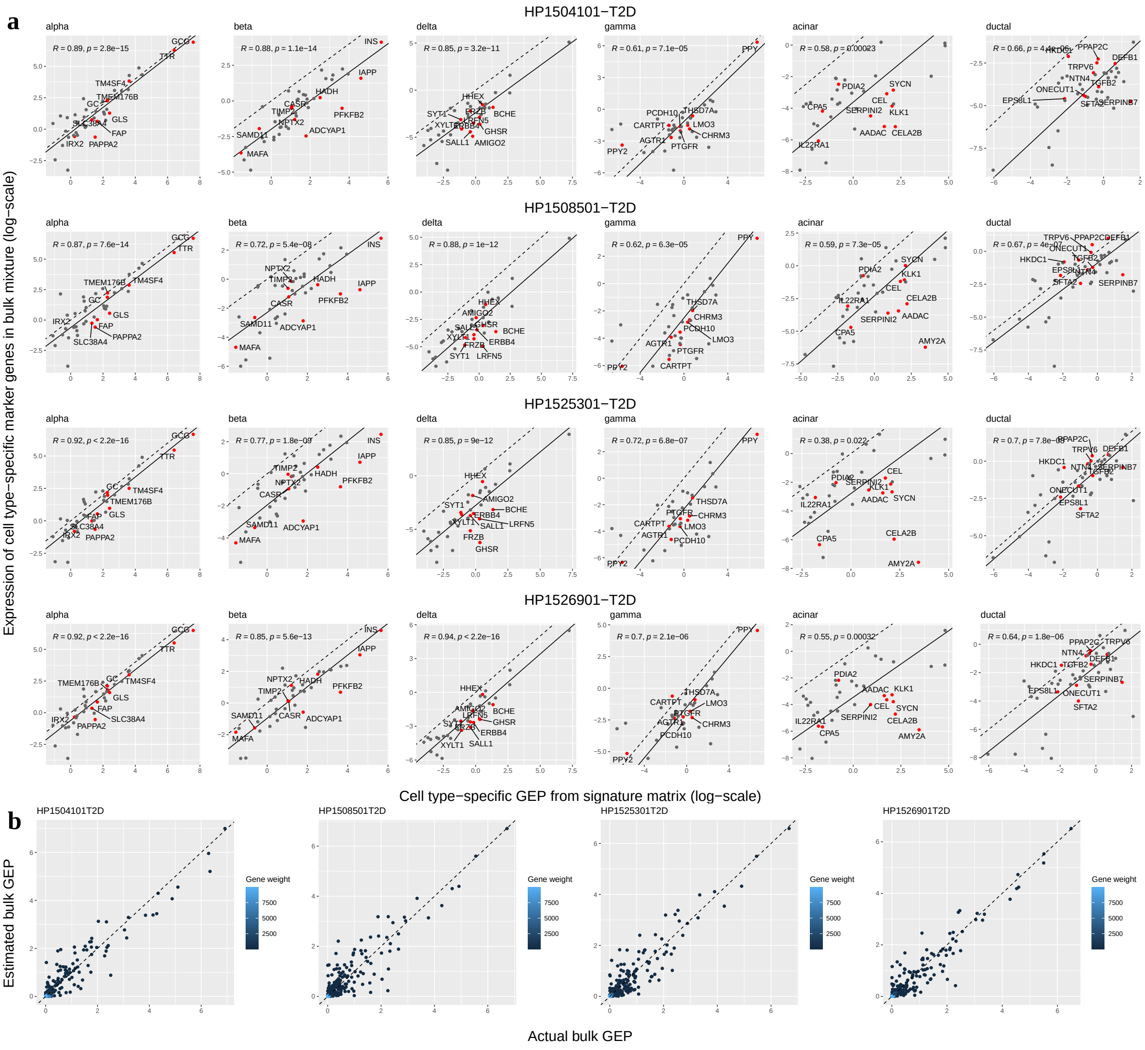

Figure S7 Sectional linear relationship between individual bulk mixtures and the signature matrix for T2D samples in the human pancreatic islet data set. a Linear regression with a slope of one for individual bulk mixtures and the cell type-specific GEPs for T2D pancreatic islet samples. The dashed line in each plot represents the line of $y = x$. The signature matrix was constructed by selecting the top 150 marker genes for each cell type. Gene symbols of the top 10 most significant marker genes were plotted. **b** Scatter plots comparing estimated and actual bulk GEPs, colored by gene weights. The dashed line in each plot represents the line of $y = x$.

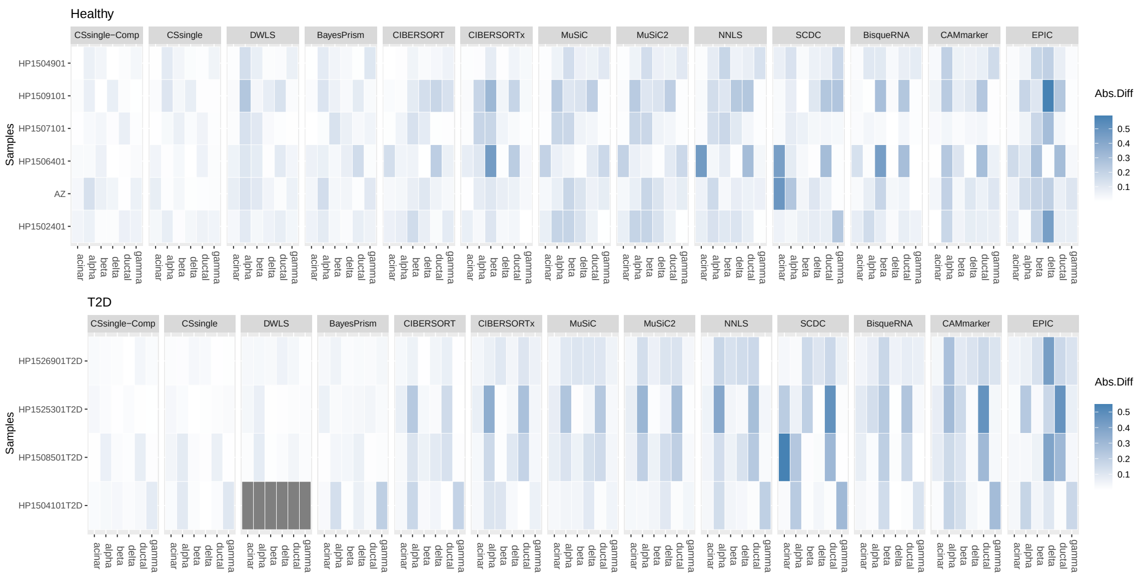

Figure S8 Decomposition benchmark in human pancreatic islet tissue. Benchmarking results are shown in heatmaps, displaying the mean absolute deviation (mAD) between true and estimated cell type proportions for healthy (top panel) and T2D (bottom panel) samples. Darker colors represent higher mAD values.

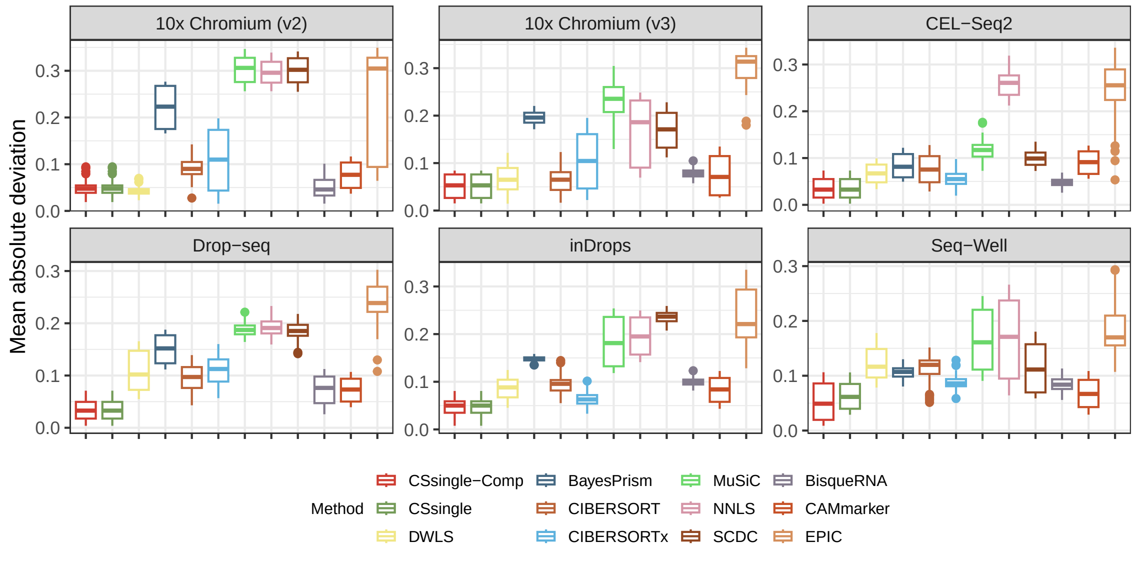

Figure S9 Decomposition benchmark in human PBMC. Benchmarking of deconvolution accuracy in terms of mean absolute deviation (mAD). Data are depicted using boxplots, where the center line indicates the median, the box boundaries signify the upper and lower quartiles, and the whiskers span the full range of maximum and minimum values.

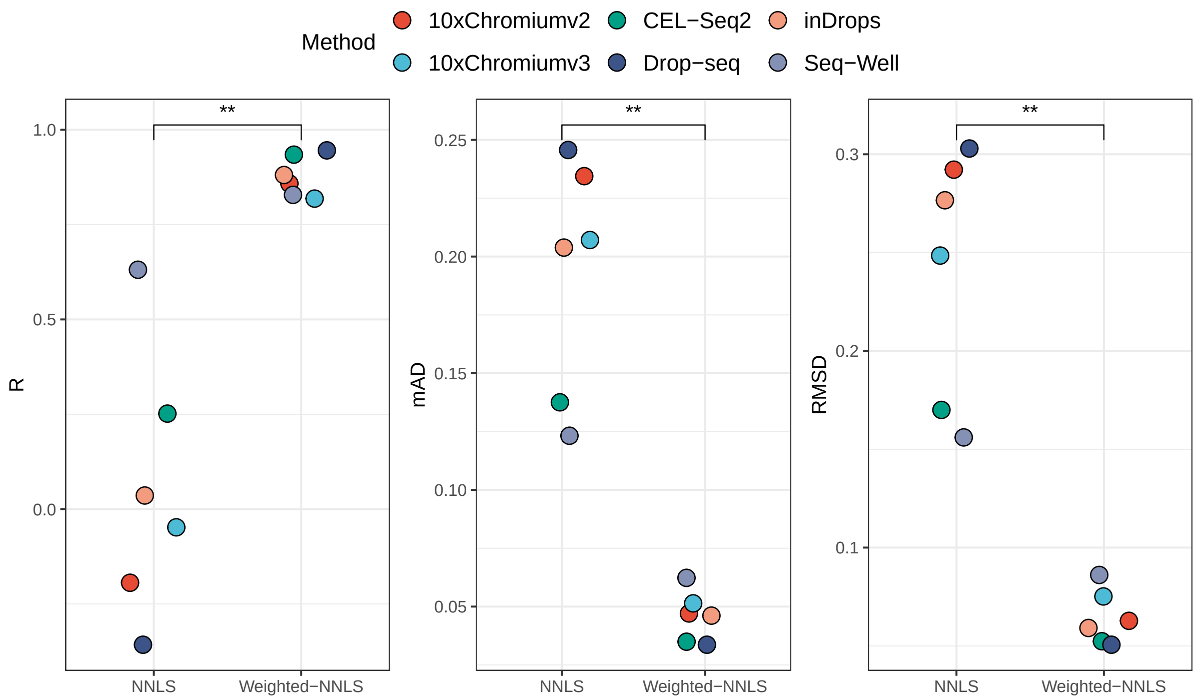

Figure S10 Comparison of the performance of CSsingle initialized with two different weighting strategies. Left panel: constant gene weights (NNLS) and Right panel: more weights on strong concordant genes and less weights on weak concordant genes (weighted-NNLS). Reported 'R' corresponds to Pearson correlation and p-values indicate the significance of these correlations.

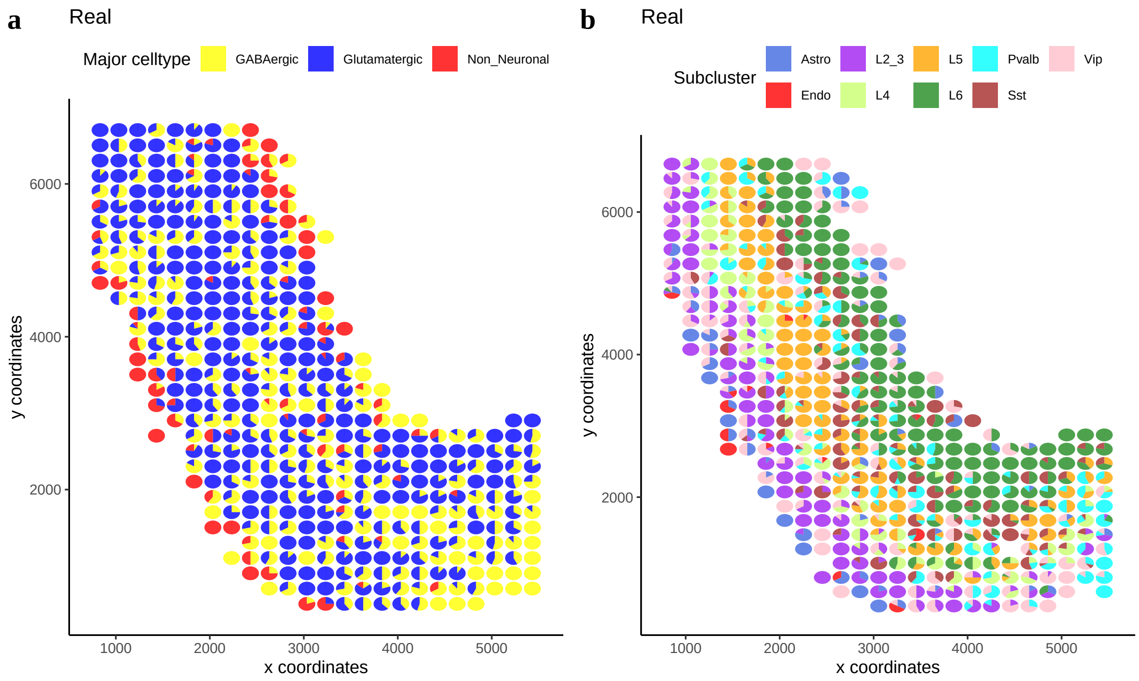

Figure S11 Scatter pie plots showing the real proportions of major (a) and minor (b) subtypes at each spot in simulated ST data of mouse brain tissue.

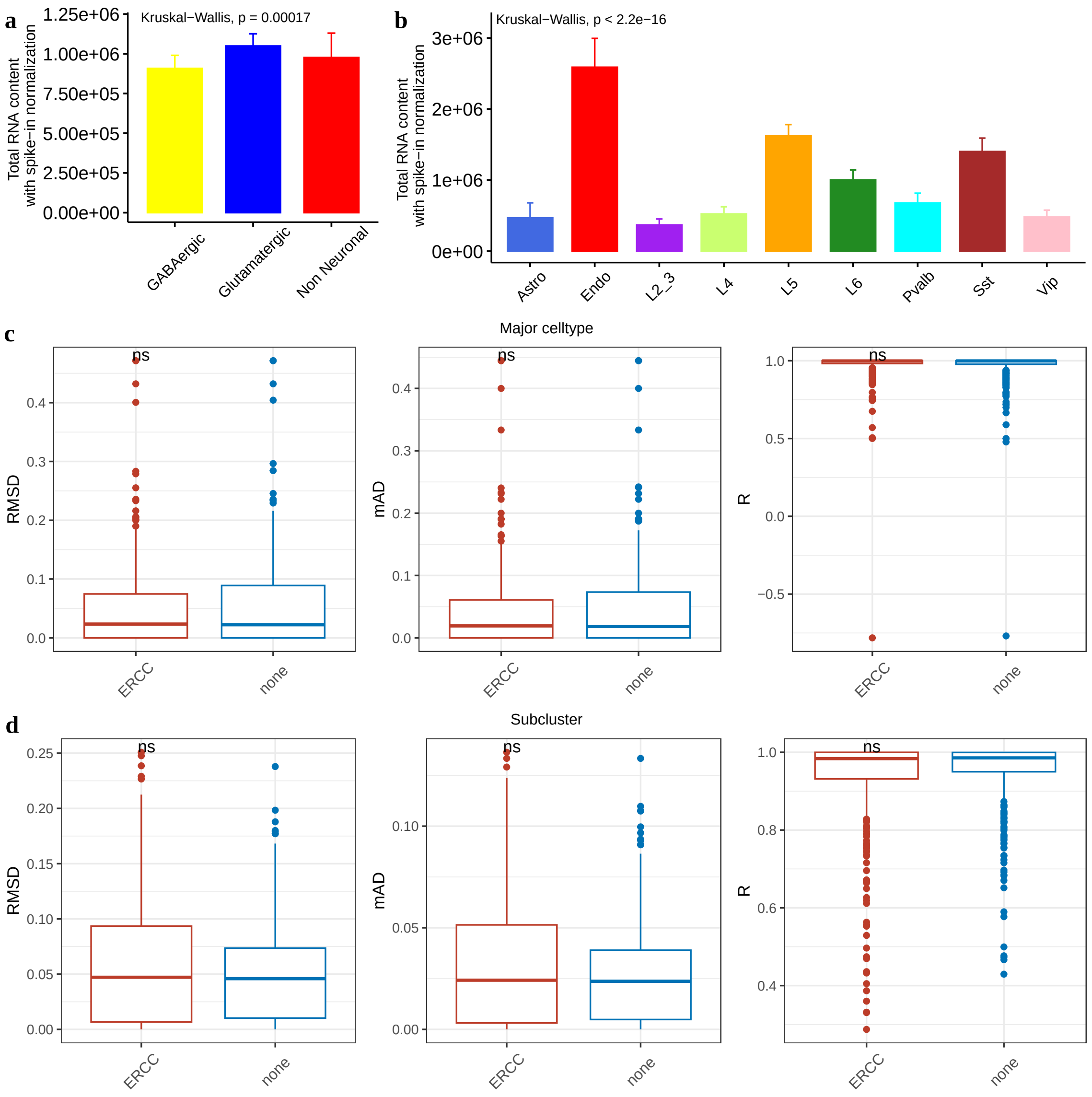

Figure S12 CSsingle-Spatial deconvolution performance in simulated ST data of mouse brain tissue: with versus without cell size correction. (**a**,**b**) Comparison of the estimated cell sizes for three major (**a**) and nine minor (**b**) subtypes. Statistical significance was assessed using Kruskal-Wallis test. (**c**,**d**) Performance comparison for major (**c**) and minor (**d**) subtypes: with versus without cell size correction. Statistical significance was assessed using a two-sided Wilcoxon test and indicated as follows: $^{ns}p \geq 0.05$.

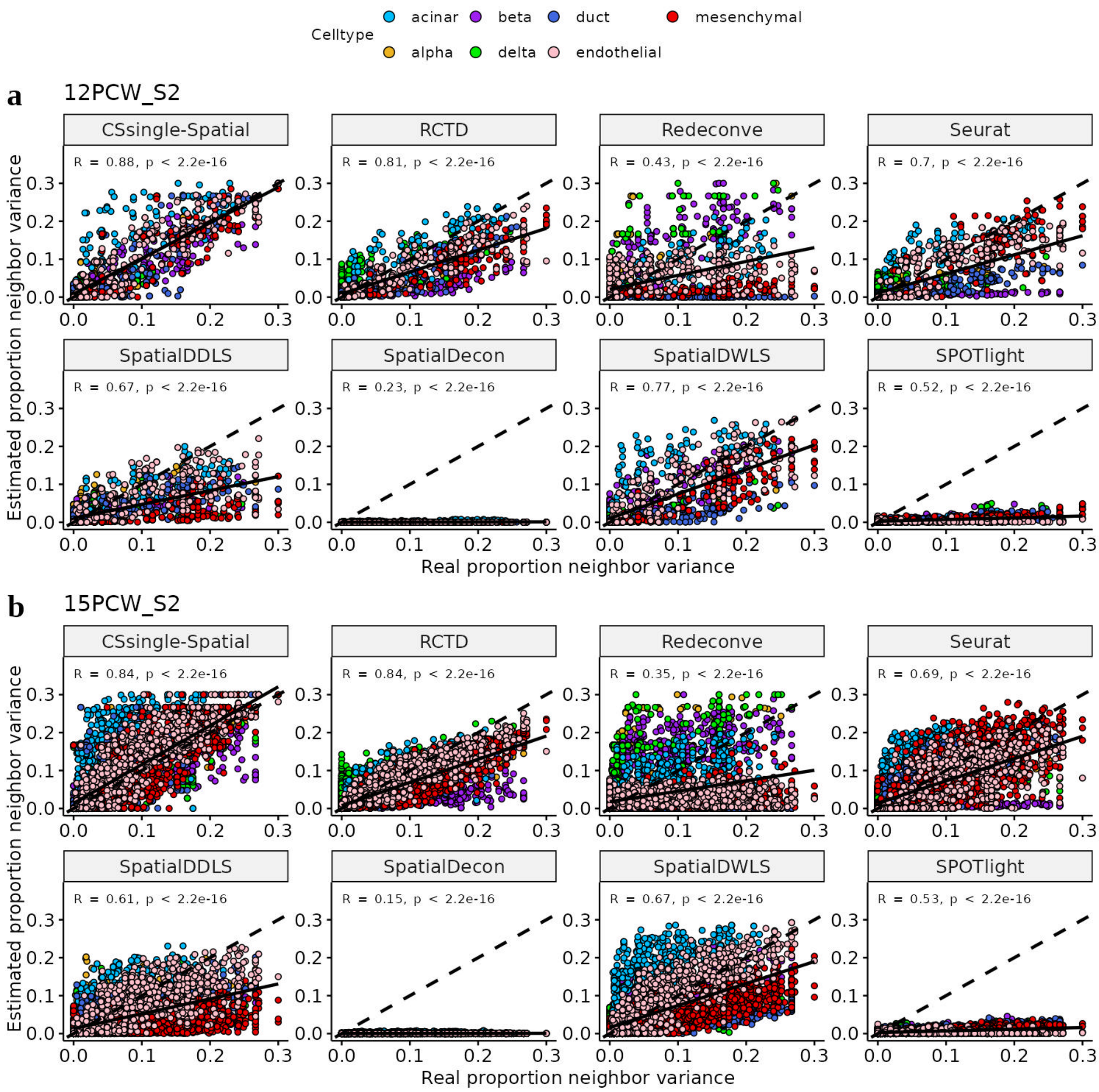

Figure S13 CSsingle-Spatial outperforms other methods in capturing local heterogeneity for (a) 12 PCW slide 2 and (b) 15 PCW slide 2.

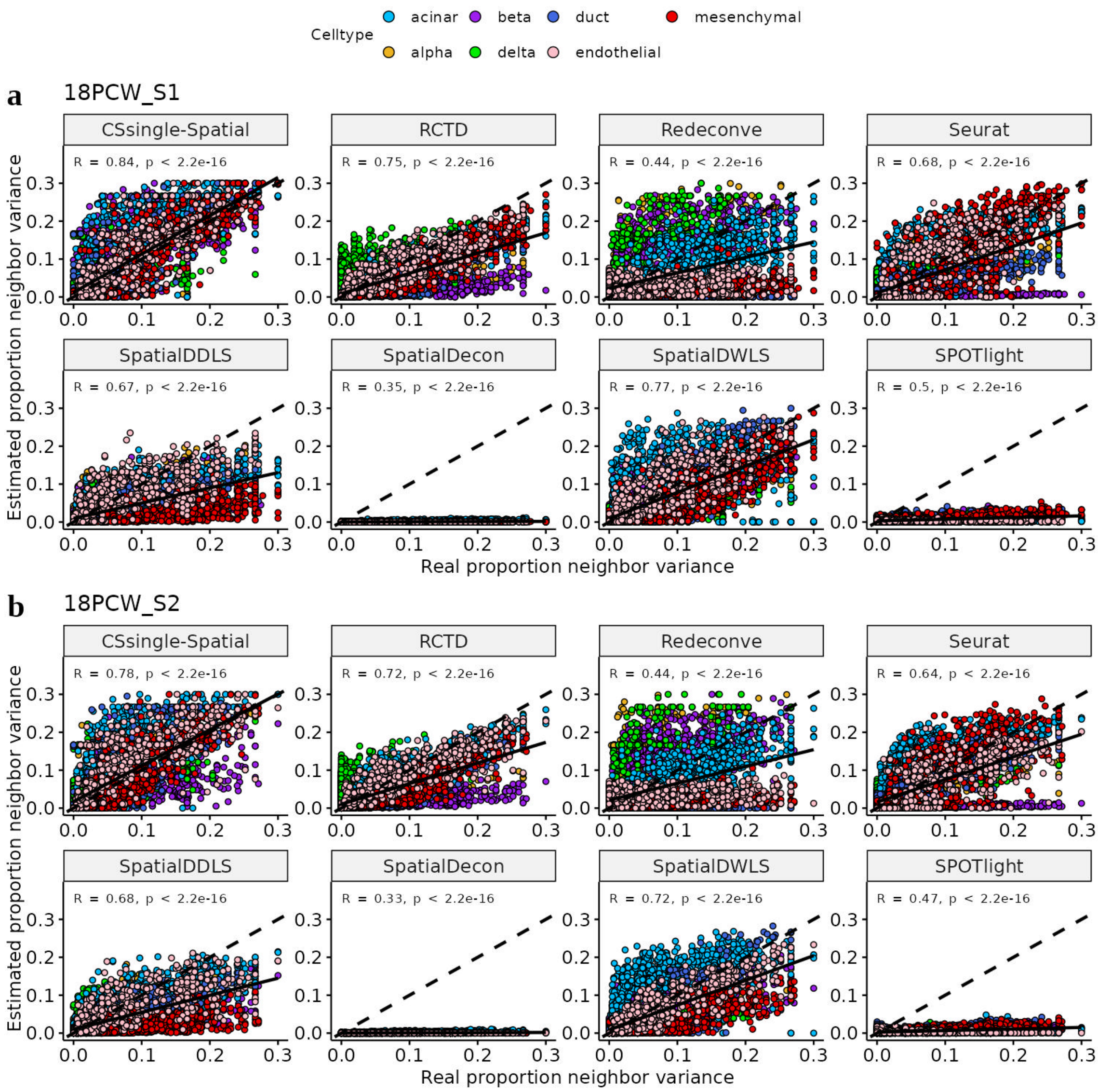

Figure S14 CSsingle-Spatial outperforms other methods in capturing local heterogeneity for (a) 18 PCW slide 1 and (b) 18 PCW slide 2.

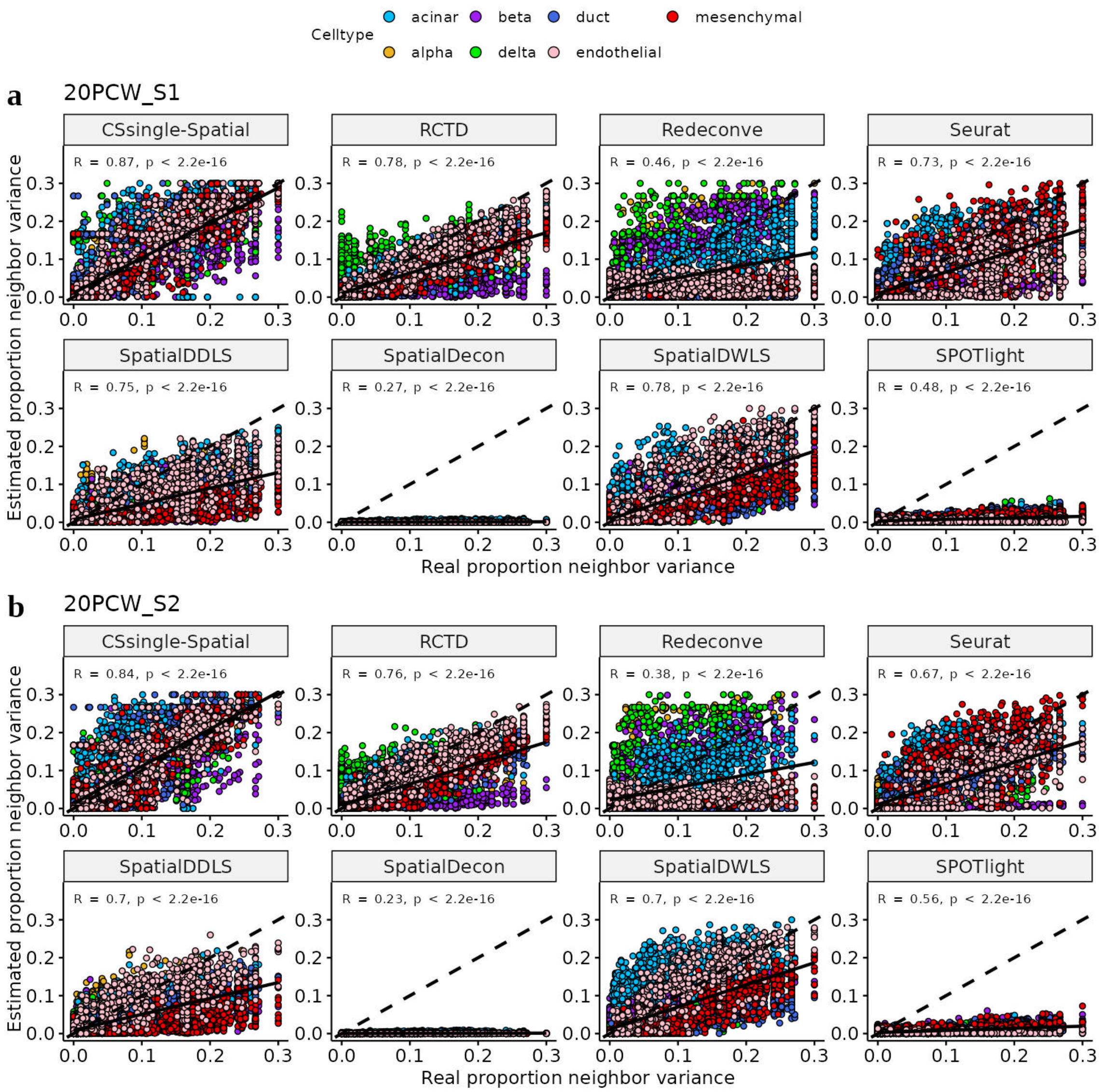

Figure S15 CSsingle-Spatial outperforms other methods in capturing local heterogeneity for (a) 20 PCW slide 1 and (b) 20 PCW slide 2.

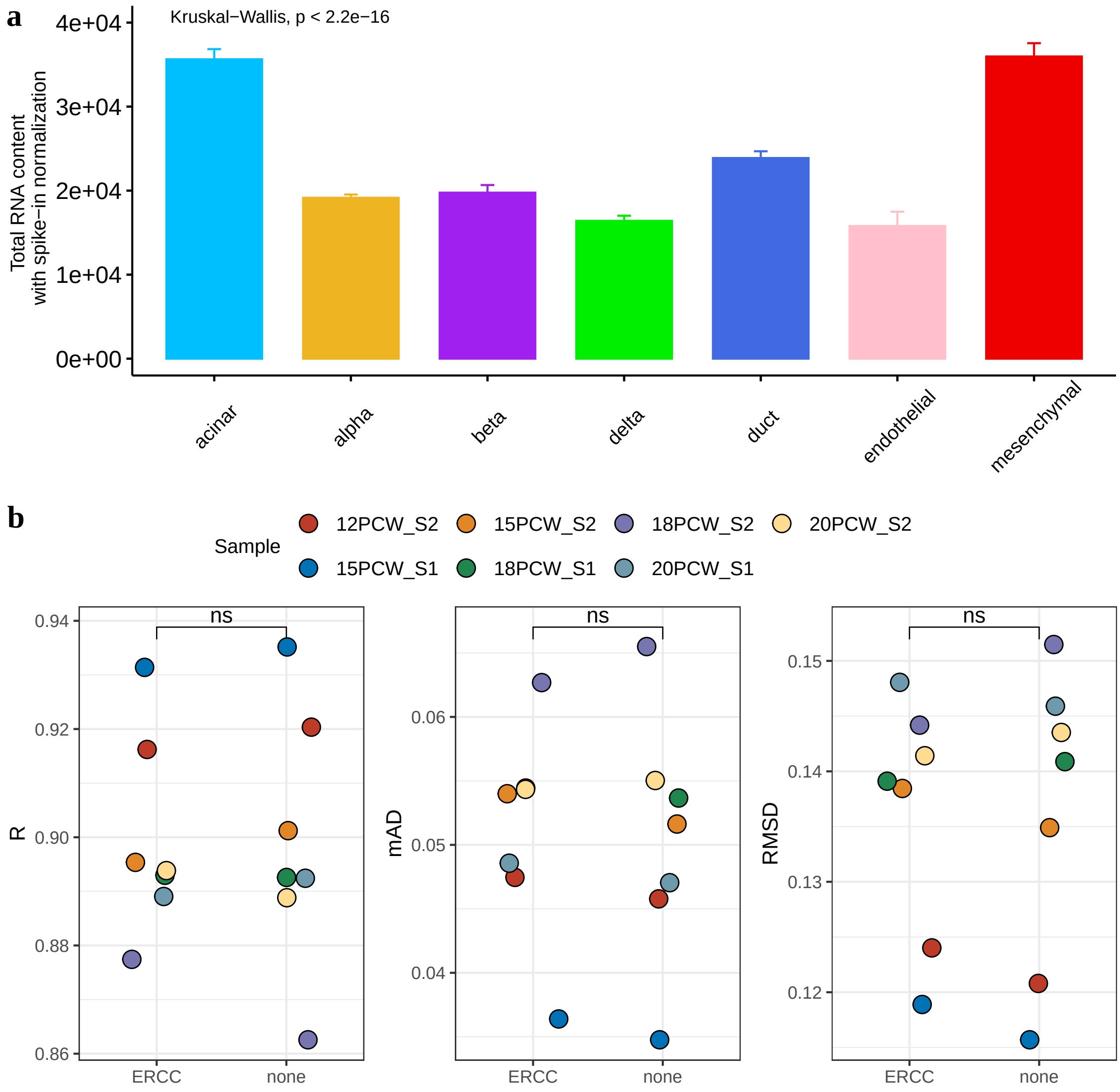

Figure S16 CSsingle-Spatial deconvolution performance in simulated ST data of human pancreas: with versus without cell size correction. (**a**) Comparison of the estimated cell sizes for seven cell types. (**b**) Comparative evaluation of deconvolution performance: with versus without cell size correction. Points are color-coded by sample origin.

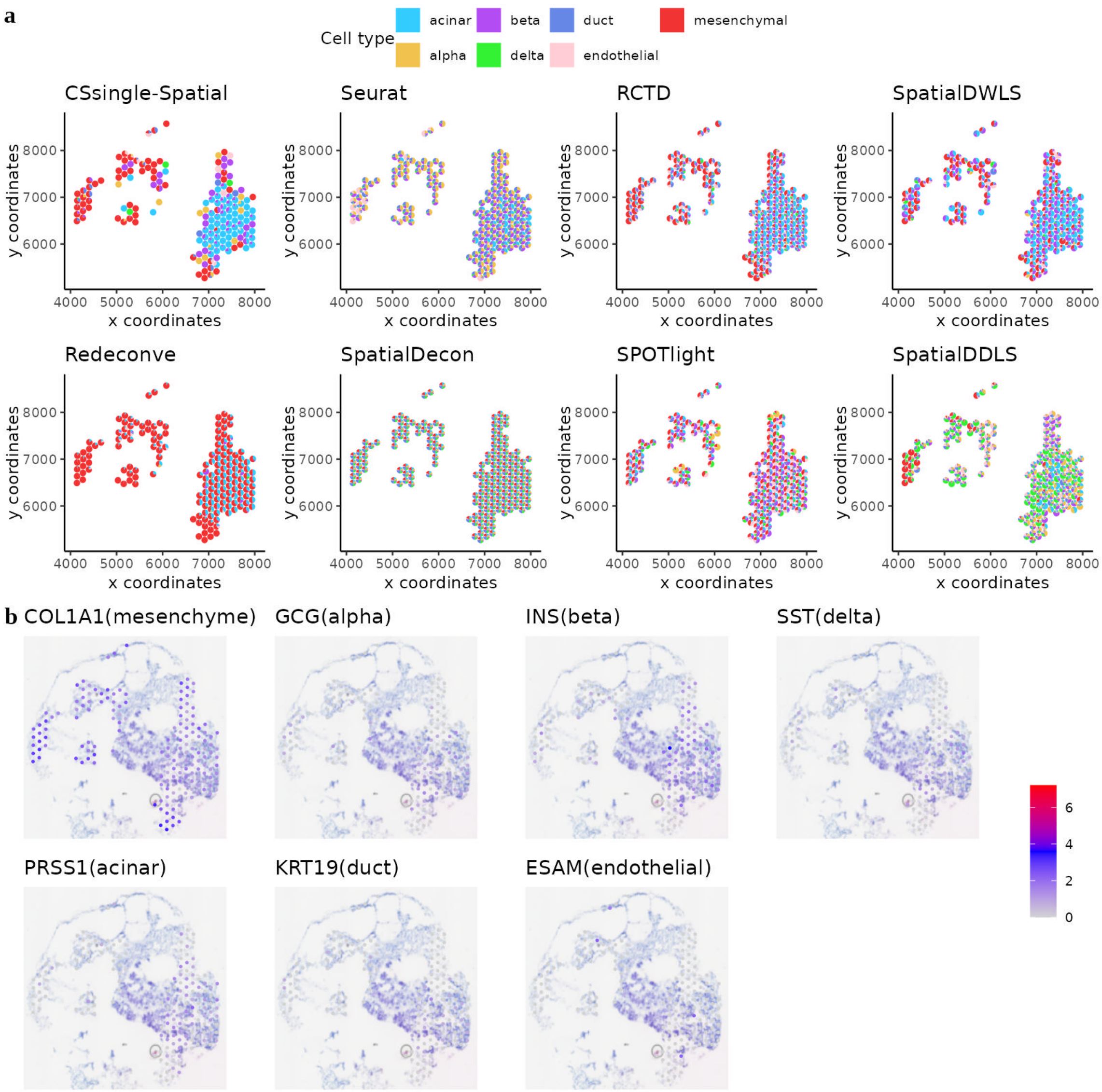

Figure S17 Decomposition benchmark on tissue section 2 from human fetal pancreas at 12 PCW. a Scatter pie plots showing the estimated cellular composition. **b** Feature plots showing some selected marker genes that were used to annotate the cell types (listed in parentheses).

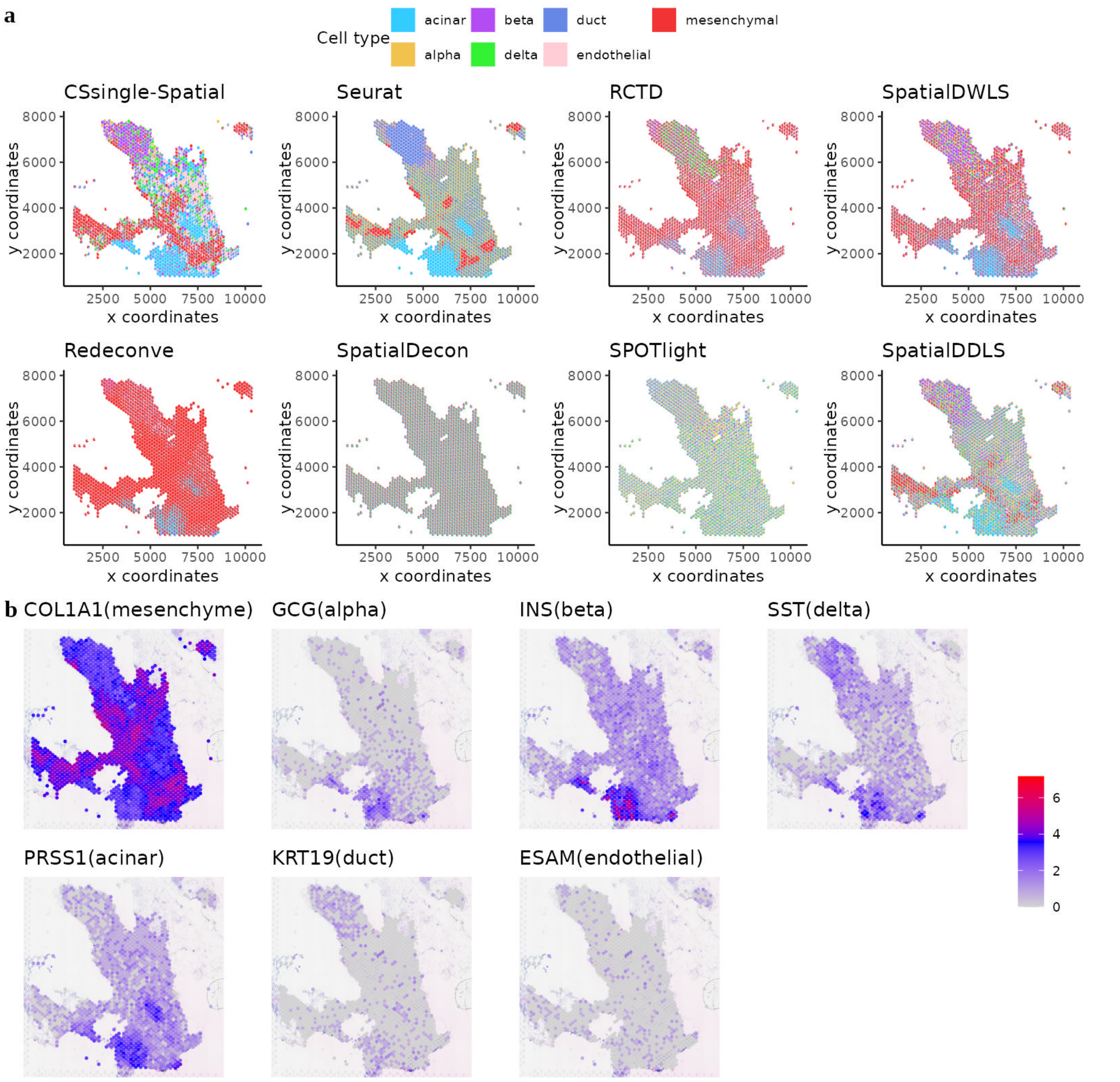

Figure S18 Decomposition benchmark on tissue section 1 from human fetal pancreas at 15 PCW. a Scatter pie plots showing the estimated cellular composition. **b** Feature plots showing some selected marker genes that were used to annotate the cell types (listed in parentheses).

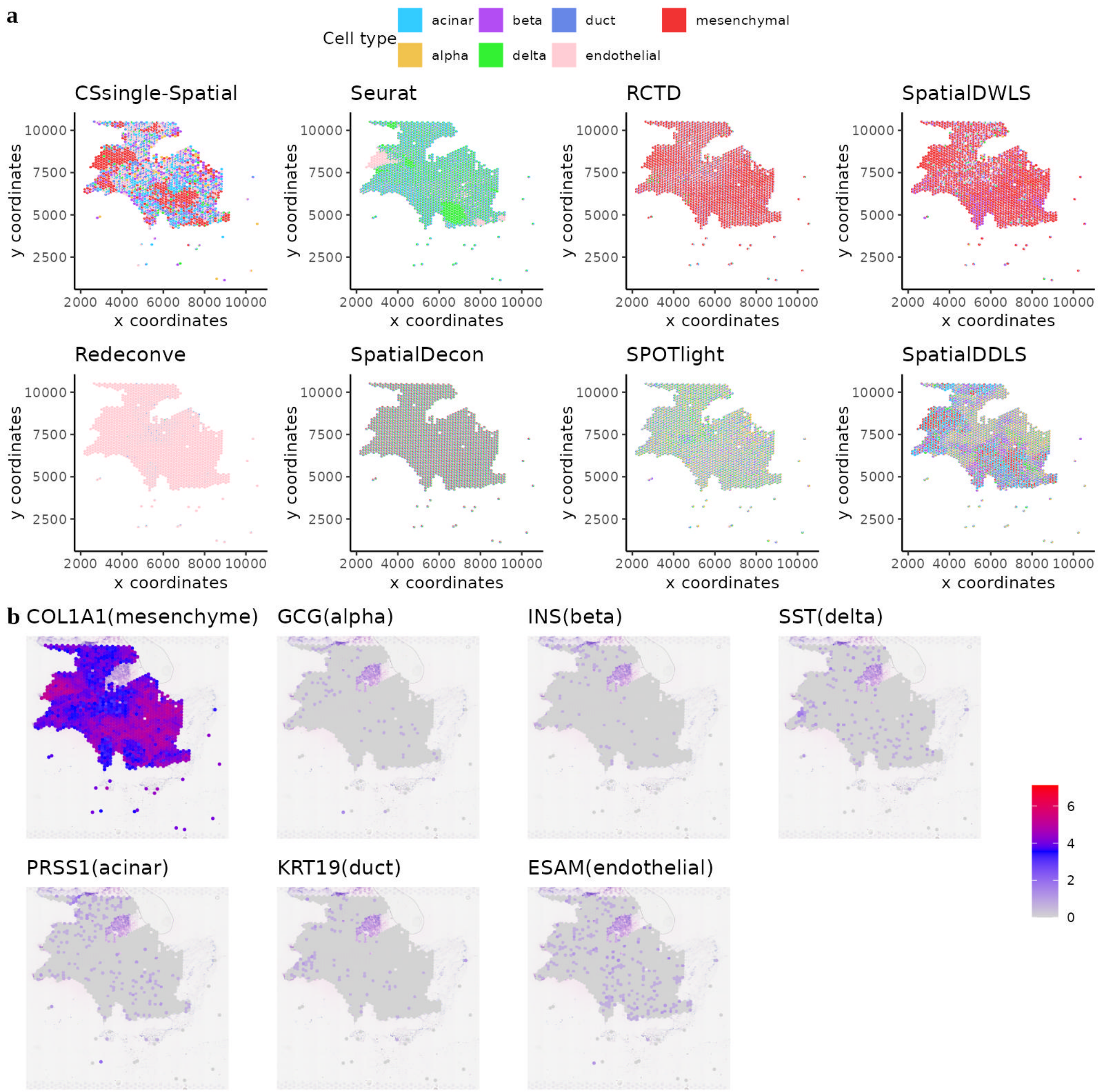

Figure S19 Decomposition benchmark on tissue section 2 from human fetal pancreas at 15 PCW. a Scatter pie plots showing the estimated cellular composition. **b** Feature plots showing some selected marker genes that were used to annotate the cell types (listed in parentheses).

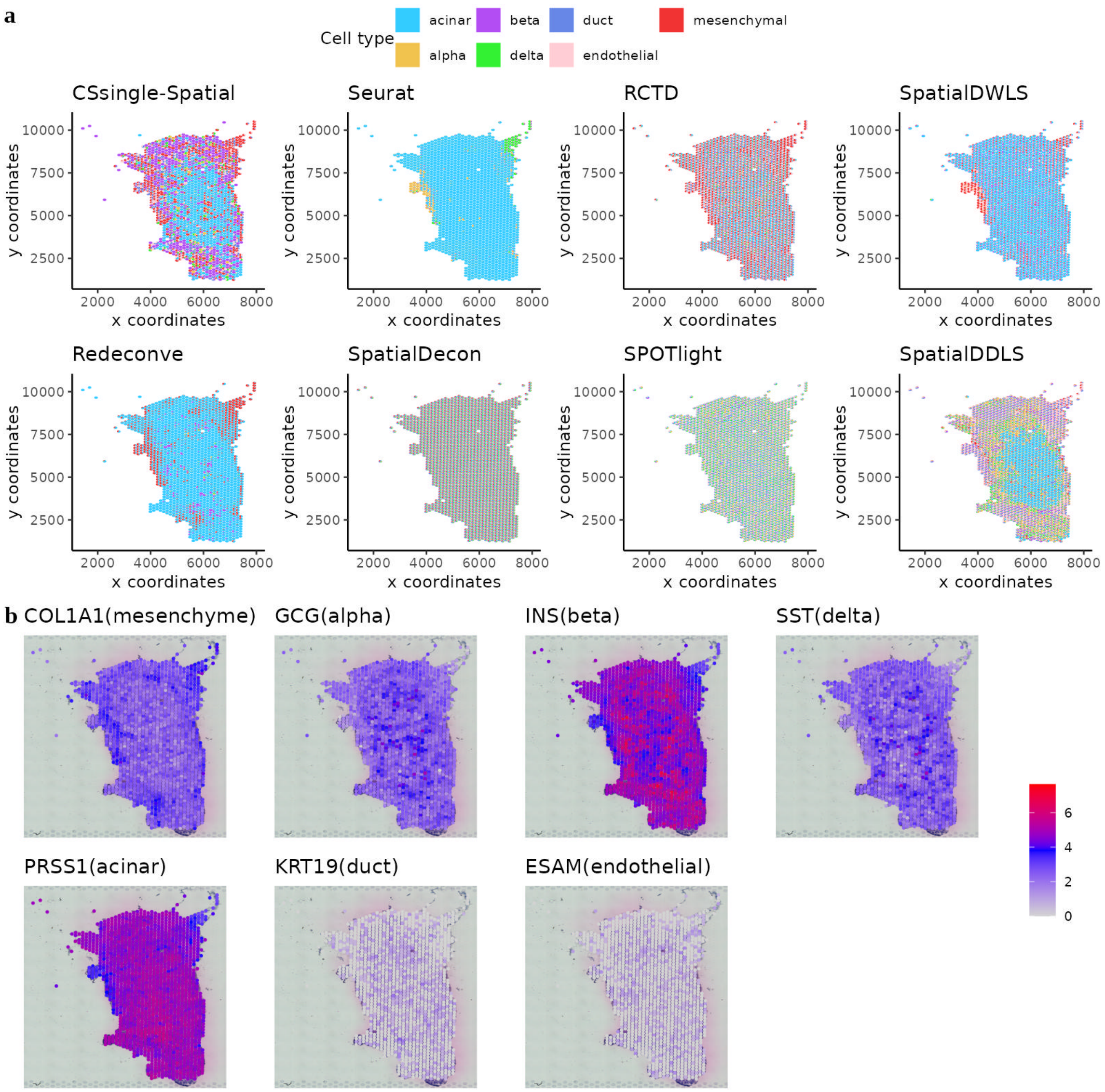

Figure S20 Decomposition benchmark on tissue section 1 from human fetal pancreas at 18 PCW. a Scatter pie plots showing the estimated cellular composition. **b** Feature plots showing some selected marker genes that were used to annotate the cell types (listed in parentheses).

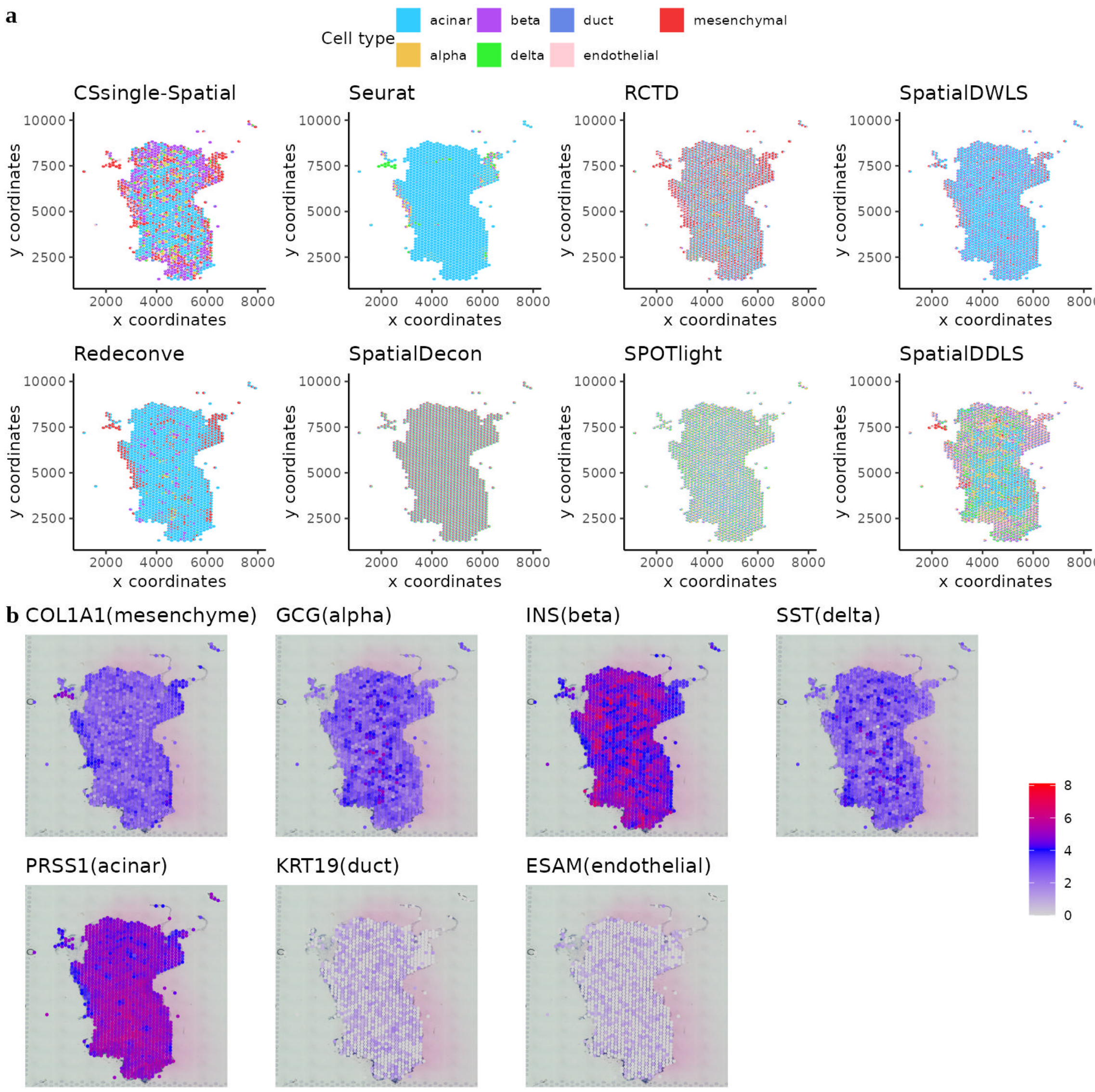

Figure S21 Decomposition benchmark on tissue section 2 from human fetal pancreas at 18 PCW. a Scatter pie plots showing the estimated cellular composition. **b** Feature plots showing some selected marker genes that were used to annotate the cell types (listed in parentheses).

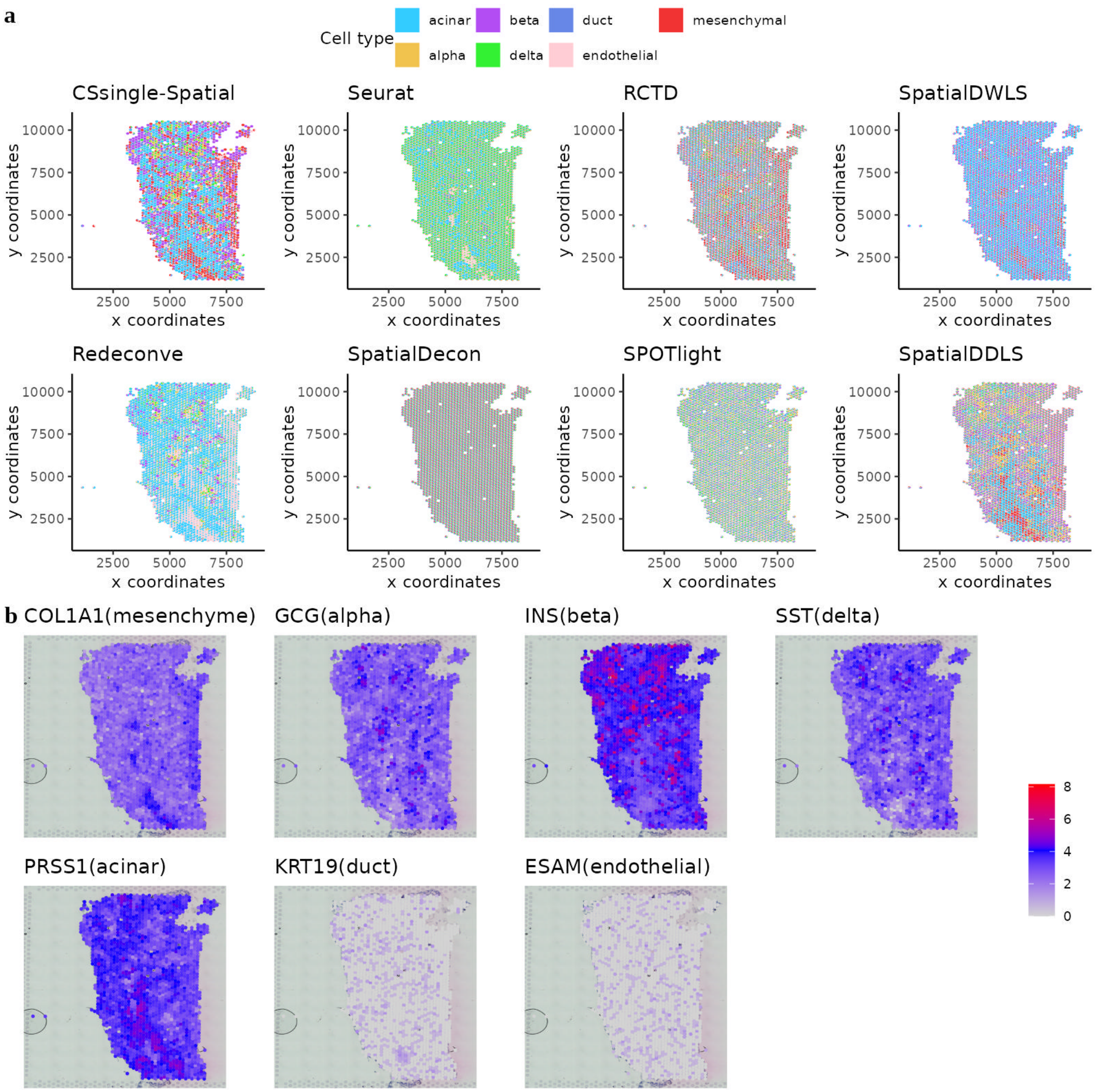

Figure S22 Decomposition benchmark on tissue section 1 from human fetal pancreas at 20 PCW. a Scatter pie plots showing the estimated cellular composition. **b** Feature plots showing some selected marker genes that were used to annotate the cell types (listed in parentheses).

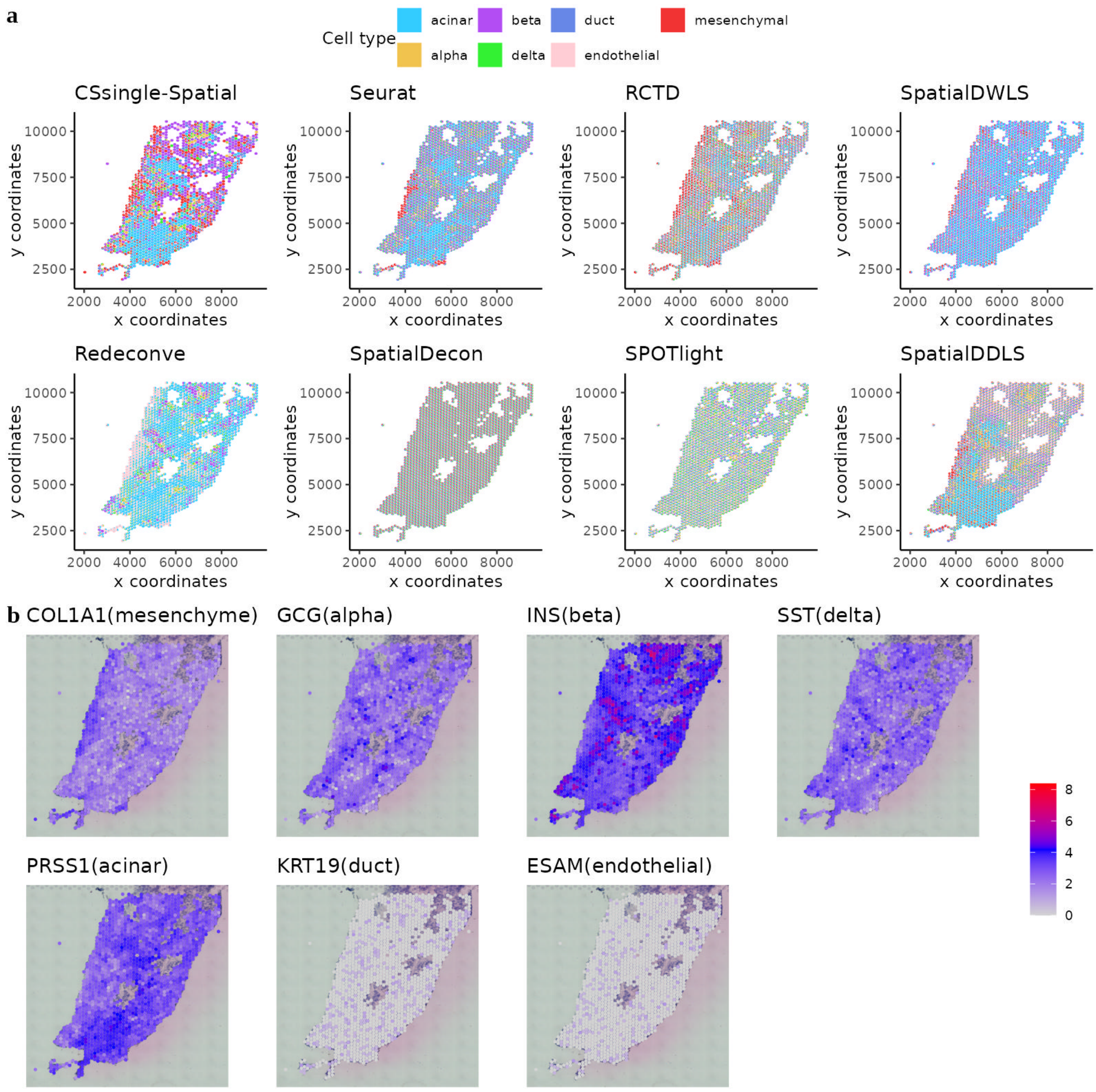

Figure S23 Decomposition benchmark on tissue section 2 from human fetal pancreas at 20 PCW. a Scatter pie plots showing the estimated cellular composition. **b** Feature plots showing some selected marker genes that were used to annotate the cell types (listed in parentheses).

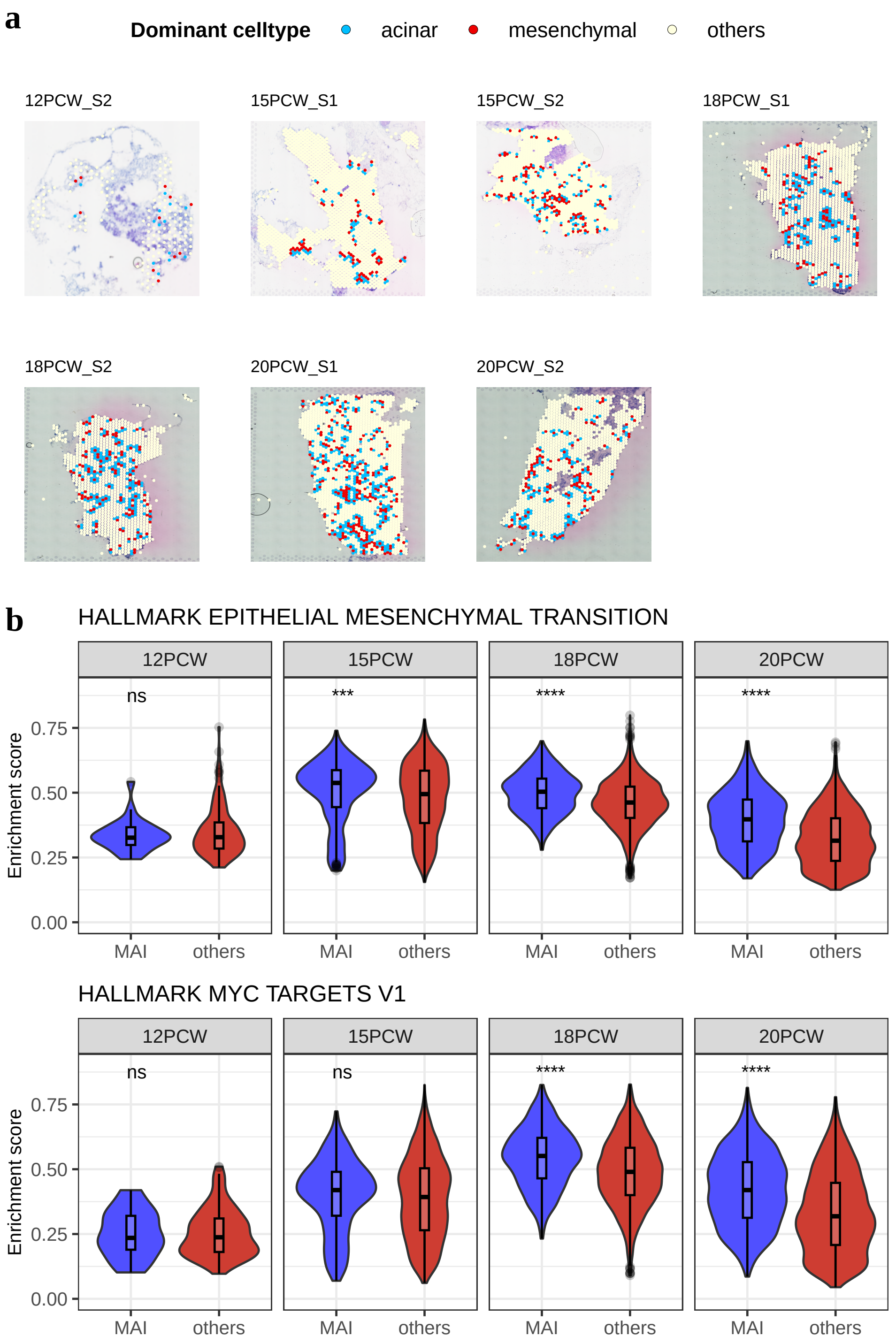

Figure S24 CSsingle-Spatial identifies spatiotemporal mesenchymal–acinar interactions (MAIs) in human pancreas development. **a** Spatial mapping of MAIs by CSsingle-Spatial reveals increased interactions at 18 and 20 PCWs. **b** Enrichment of Hallmark gene sets in MAI-associated spots versus non-MAI spots, highlighting elevated activity of epithelial–mesenchymal transition (EMT) and MYC target pathways in MAI regions at 18 and 20 PCWs.

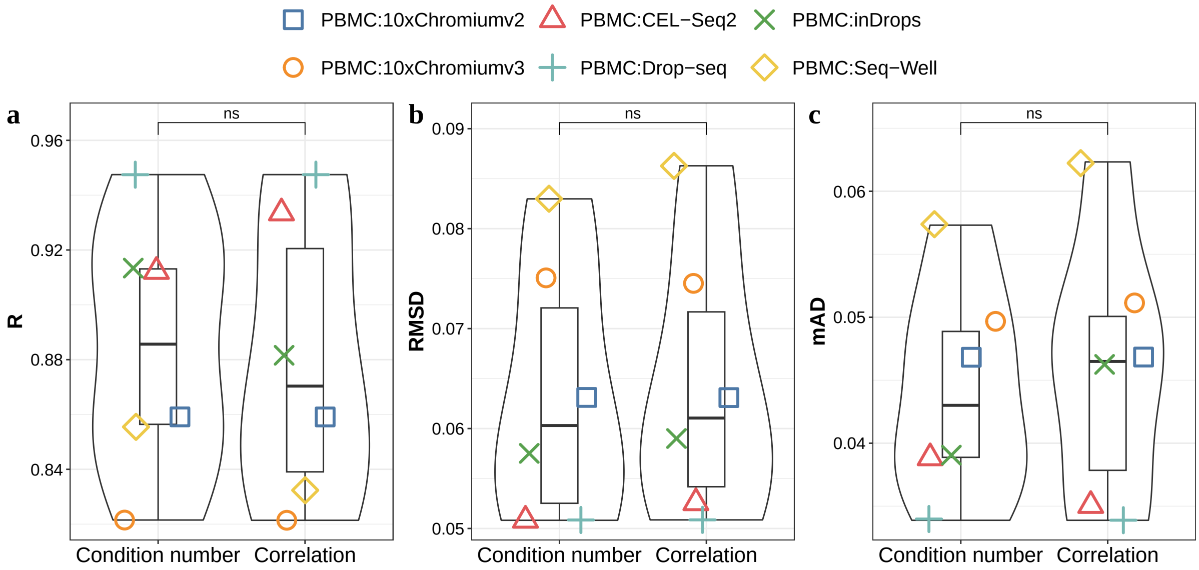

Figure S25 Comparisons of the deconvolution results by integrating CSsingle with the signature matrix generated by different strategies: condition number versus spearman correlation. The evaluation plot depicting Pearson correlation (**a**), root mean square deviation (**b**) and mean absolute deviation (**c**). Multiple signature matrices (six in total) were created by varying the number of marker genes from 50 to 300 with a step of 50 for each cell type. Nine data sets were denoted by different shapes and colors.

Table S1. Decomposition benchmark in human pancreatic islet tissue in terms of root mean square deviation (RMSD), mean absolute deviation (mAD) and Pearson correlation (R).

Method	RMSD	mAD	R
CSsingle-Comp	0.041	0.030	0.975
CSsingle	0.043	0.031	0.975
DWLS	0.071	0.052	0.952
BayesPrism	0.066	0.051	0.938
CIBERSORT	0.089	0.067	0.878
CIBERSORTx	0.125	0.084	0.755
MuSiC	0.112	0.090	0.855
MuSiC2	0.120	0.094	0.844
NNLS	0.141	0.105	0.793
SCDC	0.178	0.120	0.583
BisqueRNA	0.122	0.082	0.798
CAMmarker	0.145	0.108	0.799
EPIC	0.189	0.137	0.522

Table S2. Decomposition benchmark in human PBMC in terms of root mean square deviation (RMSD), mean absolute deviation (mAD) and Pearson correlation (R).

10x Chromium (v2)	RMSD	mAD	R	Drop-seq	RMSD	mAD	R
Cssingle-Comp	0.063	0.047	0.859	Cssingle-Comp	0.051	0.034	0.948
CSsingle	0.063	0.047	0.859	CSsingle	0.051	0.034	0.948
DWLS	0.054	0.042	0.904	DWLS	0.141	0.109	0.377
BayesPrism	0.280	0.222	-0.382	BayesPrism	0.182	0.150	0.373
CIBERSORT	0.115	0.092	0.834	CIBERSORT	0.115	0.096	0.600
CIBERSORTx	0.156	0.108	0.477	CIBERSORTx	0.140	0.110	0.375
MuSiC	0.372	0.302	-0.459	MuSiC	0.245	0.188	-0.052
NNLS	0.333	0.296	-0.300	NNLS	0.248	0.193	-0.117
SCDC	0.374	0.301	-0.457	SCDC	0.244	0.187	-0.092
BisqueRNA	0.069	0.050	0.828	BisqueRNA	0.084	0.073	0.743
CAMmarker	0.099	0.077	0.626	CAMmarker	0.091	0.072	0.706
EPIC	0.354	0.250	-0.108	EPIC	0.280	0.242	-0.531
10x Chromium (v3)	RMSD	mAD	R	inDrops	RMSD	mAD	R
Cssingle-Comp	0.075	0.051	0.821	Cssingle-Comp	0.059	0.046	0.882
CSsingle	0.075	0.051	0.821	CSsingle	0.059	0.046	0.882
DWLS	0.084	0.067	0.745	DWLS	0.113	0.085	0.837
BayesPrism	0.212	0.196	0.005	BayesPrism	0.159	0.148	0.178
CIBERSORT	0.084	0.063	0.848	CIBERSORT	0.112	0.094	0.553
CIBERSORTx	0.142	0.103	0.525	CIBERSORTx	0.079	0.063	0.797
MuSiC	0.280	0.232	-0.454	MuSiC	0.208	0.185	0.643
NNLS	0.218	0.162	0.059	NNLS	0.232	0.196	0.278
SCDC	0.200	0.170	-0.139	SCDC	0.285	0.235	-0.201
BisqueRNA	0.091	0.078	0.698	BisqueRNA	0.129	0.099	0.421
CAMmarker	0.093	0.074	0.796	CAMmarker	0.103	0.083	0.589
EPIC	0.398	0.303	-0.181	EPIC	0.305	0.243	-0.247
CEL-Seq2	RMSD	mAD	R	Seq-Well	RMSD	mAD	R
Cssingle-Comp	0.053	0.035	0.934	Cssingle-Comp	0.077	0.053	0.845
CSsingle	0.053	0.035	0.934	CSsingle	0.086	0.062	0.832
DWLS	0.094	0.067	0.745	DWLS	0.158	0.123	0.742
BayesPrism	0.107	0.084	0.680	BayesPrism	0.129	0.106	0.764
CIBERSORT	0.095	0.075	0.643	CIBERSORT	0.129	0.114	0.774
CIBERSORTx	0.064	0.055	0.861	CIBERSORTx	0.109	0.087	0.693
MuSiC	0.138	0.116	0.385	MuSiC	0.220	0.165	0.673
NNLS	0.347	0.258	0.072	NNLS	0.228	0.165	0.643
SCDC	0.122	0.099	0.488	SCDC	0.151	0.114	0.703
BisqueRNA	0.059	0.048	0.885	BisqueRNA	0.110	0.084	0.585
CAMmarker	0.120	0.090	0.509	CAMmarker	0.088	0.067	0.797
EPIC	0.315	0.252	-0.047	EPIC	0.236	0.188	0.165

Table S3. Performance of CSsingle and other methods in the human PBMC. The real proportions of cell type in the bulk data are shown in parentheses. The RMSE, mAD and R values were averaged across six different scRNA-seq methods. The smallest RMSD and mAD, and the largest R values for each cell type are highlighted in bold.

**B cells(0.152)**	RMSD	mAD	R	**Monocytes(0.152)**	RMSD	mAD	R
CSsingle-Comp	**0.02**	**0.02**	0.98	CSsingle-Comp	**0.02**	**0.02**	0.95
CSsingle	**0.02**	**0.02**	0.98	CSsingle	0.03	**0.02**	0.95
DWLS	0.04	0.04	**0.99**	DWLS	0.1	0.09	0.96
BayesPrism	0.06	0.06	**0.99**	BayesPrism	0.14	0.13	0.96
CIBERSORT	0.09	0.08	0.95	CIBERSORT	0.07	0.06	0.87
CIBERSORTx	0.07	0.07	0.95	CIBERSORTx	0.08	0.07	0.92
MuSiC	0.14	0.13	0.61	MuSiC	0.1	0.09	0.83
NNLS	0.14	0.13	-0.19	NNLS	0.22	0.2	0.69
SCDC	0.13	0.12	0.64	SCDC	0.08	0.07	0.8
BisqueRNA	0.06	0.05	0.56	BisqueRNA	0.09	0.09	0.05
CAMmarker	0.06	0.06	0.99	CAMmarker	0.03	0.03	**0.97**
EPIC	0.16	0.13	0.58	EPIC	0.46	0.4	0.54
**T cells CD4+(0.220)**	RMSD	mAD	R	**NK cells(0.092)**	RMSD	mAD	R
CSsingle-Comp	**0.04**	**0.04**	0.4	CSsingle-Comp	0.09	0.07	0.88
CSsingle	**0.04**	**0.04**	0.41	CSsingle	0.09	0.07	0.88
DWLS	0.12	0.11	-0.39	DWLS	**0.05**	**0.04**	0.96
BayesPrism	0.16	0.15	0.32	BayesPrism	0.28	0.26	**0.99**
CIBERSORT	0.12	0.11	-0.47	CIBERSORT	0.1	0.08	0.77
CIBERSORTx	0.16	0.13	-0.56	CIBERSORTx	**0.05**	**0.04**	0.96
MuSiC	0.17	0.17	-0.04	MuSiC	0.38	0.37	0.33
NNLS	0.27	0.26	0.02	NNLS	0.22	0.2	0.55
SCDC	0.15	0.15	0.24	SCDC	0.42	0.41	0.58
BisqueRNA	0.08	0.07	**0.59**	BisqueRNA	0.07	0.06	0.94
CAMmarker	0.12	0.12	0.52	CAMmarker	0.08	0.06	0.98
EPIC	0.21	0.21	-0.03	EPIC	0.22	0.19	0.11
**T cells CD8+(0.384)**	RMSD	mAD	R				
CSsingle-Comp	**0.08**	**0.07**	0.79				
CSsingle	0.09	**0.07**	0.79				
DWLS	0.13	0.12	0.9				
BayesPrism	0.19	0.17	0.91				
CIBERSORT	0.13	0.11	**0.96**				
CIBERSORTx	0.14	0.12	**0.96**				
MuSiC	0.3	0.28	-0.1				
NNLS	0.32	0.29	-0.61				
SCDC	0.24	0.22	-0.08				
BisqueRNA	0.11	0.09	0.53				
CAMmarker	0.14	0.11	0.79				
EPIC	0.35	0.32	0.04				

Table S4. Runtime efficiency and deconvolution accuracy for different step sizes used in the signature matrix construction. Multiple signature matrices were created by varying the number of marker gens from 50 to 200 with step 50 (six in total) or 1 (151 in total) for each cell type. All datasets were run on a 2.59 GHz Intel Xeon Processor with 256 GB of RAM and 32 cores. R's time module was used to obtain runtime measurements in seconds.

			step size = 50					step size = 1		
Datasets (# samples)	RMSD	mAD	R	Optimal #	Runtime(s)	RMSD	mAD	R	Optimal #	Runtime(s)
10xChromiumv2 (200)	0.06	0.05	0.86	100	1.03	0.07	0.06	0.8	76	7.58
10xChromiumv3 (200)	0.07	0.05	0.82	150	1.06	0.08	0.05	0.78	132	7.7
CEL-Seq2 (200)	0.05	0.04	0.93	200	32.24	0.05	0.03	0.94	182	448.9
Drop-seq (200)	0.05	0.03	0.95	50	31.05	0.05	0.03	0.95	50	431.31
inDrops (200)	0.06	0.05	0.88	100	58.32	0.06	0.05	0.88	75	462.1
Seq-Well (200)	0.09	0.06	0.83	100	1.39	0.09	0.07	0.82	82	8.23
